# The Effect of the *APOE*-ε4 Allele on the Cholinergic Circuitry for Subjects With Different Levels of Cognitive Impairment

**DOI:** 10.3389/fneur.2021.651388

**Published:** 2021-10-13

**Authors:** Ying-Liang Larry Lai, Kuan Chen, Tzu-Wei Lee, Chao-Wei Tso, Hui-Hsien Lin, Li-Wei Kuo, Cheng-Yu Chen, Hua-Shan Liu

**Affiliations:** ^1^Ph.D. Program for Neural Regenerative Medicine, College of Medical Science and Technology, Taipei Medical University and National Health Research Institutes, Taipei, Taiwan; ^2^School of Biomedical Engineering, College of Biomedical Engineering, Taipei Medical University, Taipei, Taiwan; ^3^Computed Tomography (CT) and Magnetic Resonance (MR) Division, Rotary Trading Co., Ltd., Taipei, Taiwan; ^4^Institute of Biomedical Engineering and Nanomedicine, National Health Research Institutes, Miaoli, Taiwan; ^5^Institute of Medical Device and Imaging, National Taiwan University College of Medicine, Taipei, Taiwan; ^6^Department of Radiology, School of Medicine, College of Medicine, Taipei Medical University, Taipei, Taiwan; ^7^Department of Medical Imaging, Taipei Medical University Hospital, Taipei, Taiwan; ^8^International Ph.D. Program in Biomedical Engineering, College of Biomedical Engineering, Taipei Medical University, Taipei, Taiwan

**Keywords:** Alzheimer's disease, cholinergic pathway, nucleus basalis of Meynert, amygdala, *APOE*-ε4 allele

## Abstract

**Background:** Cholinergic deficiency has been suggested to associate with the abnormal accumulation of Aβ and tau for patients with Alzheimer's disease (AD). However, no studies have investigated the effect of *APOE*-ε4 and group differences in modulating the cholinergic basal forebrain–amygdala network for subjects with different levels of cognitive impairment. We evaluated the effect of *APOE*-ε4 on the cholinergic structural association and the neurocognitive performance for subjects with different levels of cognitive impairment.

**Methods:** We used the structural brain magnetic resonance imaging scans from the Alzheimer's Disease Neuroimaging Initiative dataset. The study included cognitively normal (CN, *n* = 167) subjects and subjects with significant memory concern (SMC, *n* = 96), early mild cognitive impairment (EMCI, *n* = 146), late cognitive impairment (LMCI, *n* = 138), and AD (*n* = 121). Subjects were further categorized according to the *APOE*-ε4 allele carrier status. The main effects of *APOE*-ε4 and group difference on the brain volumetric measurements were assessed. Regression analyses were conducted to evaluate the associations among cholinergic structural changes, *APOE*-ε4 status, and cognitive performance.

**Results:** We found that *APOE*-ε4 carriers in the disease group showed higher brain atrophy than non-carriers in the cholinergic pathway, while there is no difference between carriers and non-carriers in the CN group. *APOE*-ε4 allele carriers in the disease groups also exhibited a stronger cholinergic structural correlation than non-carriers did, while there is no difference between the carriers and non-carriers in the CN subjects. Disease subjects exhibited a stronger structural correlation in the cholinergic pathway than CN subjects did. Moreover, *APOE-*ε4 allele carriers in the disease group exhibited a stronger correlation between the volumetric changes and cognitive performance than non-carriers did, while there is no difference between carriers and non-carriers in CN subjects. Disease subjects exhibited a stronger correlation between the volumetric changes and cognitive performance than CN subjects did.

**Conclusion:** Our results confirmed the effect of *APOE-*ε4 on and group differences in the associations with the cholinergic structural changes that may reflect impaired brain function underlying neurocognitive degeneration in AD.

## Introduction

Cholinergic neurons exhibit selective neuronal vulnerability to Alzheimer's disease (AD) pathology, with hypofunction associated with the formation of Aβ plaques, tau pathology, and AD severity (1). Cholinergic deficiency may lead to the abnormal accumulation of Aβ and tau in cholinoreceptive cortical neurons (1), in which cholinergic receptors have a high affinity for Aβ and tau proteins (2). The cholinergic system has been suggested to mediate tau phosphorylation, which is reduced and increased by muscarinic and nicotinic acetylcholine receptors, respectively (3, 4). Because of the central role of acetylcholine in cognitive function and neuronal plasticity, its cognitive decline in AD is likely related to progressive basal forebrain presynaptic cholinergic deficits affecting central cholinergic transmission (5).

In AD, the topographies of cortical degeneration vary with the longitudinal degeneration of the basal forebrain, which closely reflects the known anatomical organization of the cholinergic projection system (1). The probabilistic cytoarchitectonic atlas maps of the basal forebrain comprise the following four major subregions of cholinergic cells (6): Ch1 belongs to the medial septal nucleus, Ch2 refers to the vertical limb of the diagonal band of the Broca area, Ch3 belongs to the horizontal part of the diagonal band of the Broca area, and Ch4 denotes the cholinergic cells in the nucleus basalis of Meynert (NBM). Specifically, the Ch4 (i.e., NBM) of the magnocellular cholinergic neurons is the main source of the cholinergic input to the amygdala (7). The amygdala is a subcortical limbic structure that is densely innervated by cholinergic neurons and is involved in the processing of memory and emotion (8). Compared with elderly healthy subjects, patients with amnestic mild cognitive impairment (MCI) exhibit a difference in the cholinergic structural correlation, specifically between the atrophy of the NBM and amygdala (7).

The *APOE*-ε4 allele is a genetic risk factor for AD, which would lower the age of AD onset in a gene dose-dependent manner (9, 10). The *APOE*-ε4 risk variant has been found to modify the association between cognitive performance and cerebral morphology in healthy middle-aged individuals (9). Studies are yet to elucidate how the specific amygdala and basal forebrain subnuclei atrophy relate to cognitive functioning, which is affected by the *APOE*-ε4 allele at different levels of cognitive impairment. Investigating the interaction between cholinergic regions and the effect of the *APOE*-ε4 allele on modulating neurocognitive performance at various disease groups can increase our understanding of the dynamic interplay in disease progression.

To the best of our knowledge, the effects of the *APOE*-ε4 allele on the structural association in the cholinergic circuitry and its correlation with neurocognitive performance for subjects with different levels of cognitive impairment have not been examined. Therefore, we evaluated the effects of the *APOE*-ε4 allele on the cholinergic structural association and neurocognitive performance in groups with different levels of cognitive impairment. In comparison with healthy control subjects, we hypothesized the presence of abnormality in the cholinergic pathway with its aberrant structural association and cognitive performance as modulated by the *APOE*-ε4 allele for patients at different disease groups.

## Materials and Methods

### Subjects

Data used in this article were obtained from the Alzheimer's Disease Neuroimaging Initiative (ADNI) database (adni.loni.usc.edu) (11). The ADNI was launched in 2003 as a public–private partnership, led by the principal investigator, Michael W. Weiner, MD. The primary goal of ADNI has been to test whether serial magnetic resonance imaging (MRI), positron emission tomography (PET), other biological markers, and clinical and neuropsychological assessment can be combined to measure the progression of MCI and early AD. In the present study, MRI data along with the demographic information, neurocognitive assessments, and validated composite scores derived from global cognitive composite scores at baseline were downloaded from the ADNI-2 database. This study was approved by the local ethics committees from all participating institutions, and all participants gave written informed consent. ADNI global cognitive composite scores, including memory (MEM), executive functioning (EF), language (LAN), and visuospatial functioning (VS) at baseline have been described in detail in previous studies (12, 13). We only included subjects with an image quality that passed the image quality control as described in the section of *Data Analyses*. Subjects with cognitively normal (CN, *n* = 167), significant memory concern (SMC, *n* = 96), early MCI (EMCI, *n* = 146), late cognitive impairment (LMCI, *n* = 138), and AD (*n* = 121) were included. Subjects were further categorized according to the *APOE*-ε4 allele carrier status. APOE genotyping is described in detail at http://www.adni-info.org.

### MRI Data

High-resolution T1 structural MRI data acquired through 3 Tesla MR scanners were downloaded (see Supplementary Table S1). The details of MRI protocols are listed in the ADNI website (http://adni.loni.usc.edu/methods/documents/mri-protocols/).

### Data Analyses

Region-of-interest (ROI) masks were created using the SPM Anatomy toolbox (14) with cytoarchitectonic probability anatomical maps (15, 16). The ROI masks included the Ch123, NBM, and amygdala covering both left and right hemispheres. Voxel-based morphometry (VBM) analyses were performed using the CAT12 toolbox (17, 18). Preprocessing included full iterative SPM bias correction; normalization to the standard Montreal Neurological Institute template using the diffeomorphic anatomical registration through an exponentiated lie algebra algorithm; and segmentation into gray matter (GM), white matter (WM), and cerebrospinal fluid (CSF). Smoothening of the normalized GM images was performed using a Gaussian filter (4-mm full-width half-maximum). Quality control was performed to eliminate potential outliers. We used the Check Sample Homogeneity tool for VBM data from the CAT12 toolbox to check the quality of the images for subsequent analyses (19). After the correlation between all the volumes was calculated, subjects that exhibited a correlation below 2 standard deviations and abnormalities on their segmented GM volume (e.g., images with low signal intensity, inhomogeneities, and warping errors) were not included in the subsequent data analyses (19).

### Statistical Analyses

Group differences in demographic characteristics were analyzed using one-way analysis of variance (ANOVA) and a χ^2^-test for categorical variables. A 5 (CN, SMC, EMCI, LMCI, and AD groups) × 2 (*APOE*-ε4 carriers and non-carriers) factorial analysis of covariance (ANCOVA) was conducted to investigate the main effects of the group and *APOE*-ε4 and the interaction effect of *APOE*-ε4 × group on global composite scores after controlling for age, sex, educational level (edu), and total intracranial volume (TIV = GM + WM + CSF). SPSS software (version 27.0) was used. *P* < 0.05 was considered statistically significant.

#### Group Comparison of MRI Volume Measurement

To compare volumetric measures across different groups and the *APOE*-ε4 effect, the data were modeled using 5 × 2 ANCOVA (20), resulting in the following design cells: CN(+), CN(–), SMC(+), SMC(–), EMCI(+), EMCI(–), LMCI(+), LMCI(–), AD(+), and AD(–), where (+) denotes *APOE*-ε4 carriers and (–) denotes non-carriers. Age, sex, edu, and TIV were included as nuisance covariates. We focused on the main effects of the group with different levels of cognitive impairment and the *APOE*-ε4 genotype as well as the interaction effect between the group and *APOE*-ε4 genotype. The interaction effect (*APOE*-ε4 × group) regarding the differences of how *APOE*-ε4 genotype impacts on the cholinergic brain areas between any of the four disease (SMC, EMCI, LMCI, and AD) and CN groups was accessed by the *F*-contrasts [CN vs. SMC (1 −1 −1 1 0 0 0 0 0 0), CN vs. EMCI (1 −1 0 0 −1 1 0 0 0 0), CN vs. LMCI (1 −1 0 0 0 0 −1 1 0 0), and CN vs. AD (1 −1 0 0 0 0 0 0 −1 1)] (20), following the order of [CN(+), CN(–), SMC(+), SMC(–), EMCI(+), EMCI(–), LMCI(+), LMCI(–), AD(+), and AD(–)]. Correction for multiple comparison was performed using the non-parametric threshold-free cluster enhancement (TFCE) approach with 10,000 permutations (21, 22). Statistical significance was set at *P* < 0.05 using the family-wise error (FWE) method. SPM software was used for the analysis.

#### Structural Associations Between the Amygdala and Basal Forebrain Subregions

Mean values for the significant clusters identified in ANCOVA analysis were extracted using the MarsBaR toolbox (http://marsbar.sourceforge.net). To evaluate the effect of *APOE*-ε4 on modulating the relationship between volumetric changes in the amygdala and NBM, a series of linear regression was performed in each group using the following equation (9):

Amygdala = NBM + *APOE*-ε4 + (NBM × *APOE*-ε4) + age + sex + edu + TIV

The amygdala volume was considered as the dependent variable, whereas the NBM volume; *APOE*-ε4; and interaction term between the NBM volume and *APOE*-ε4, age, sex, edu, and TIV were considered as the independent variables. The interaction term was used to test the hypothesis that the relationship between the volumetric changes in the NBM and the amygdala is different in *APOE*-ε4 carriers and non-carriers. A similar approach has been implemented in neuroimaging studies investigating the effect of the *APOE*-ε4 genotype on brain morphology (9, 23, 24).

Similarly, to assess the effect of different disease groups on modulating the relationship between the volumetric changes in the amygdala and NBM, separate linear regression analyses were conducted for each disease group using the following regression equation (9):

Amygdala = NBM + group + (NBM × group) + age + sex + edu + TIV

The amygdala volume was considered as the dependent variable, whereas the effects of the NBM volume, group (disease vs. CN groups), and the interaction term between the NBM volume and group were treated as the independent variables. The interaction term was used to test the hypothesis that the relationship between the volumetric changes in the NBM and the amygdala was different in the disease groups and the CN group. Age, sex, TIV, and edu were also added as independent variables.

#### Associations Between Volumetric Changes in the Cholinergic Regions and Neurocognitive Performance

To investigate the effect of *APOE*-ε4 on modulating the relationship between cognitive performance and volumetric changes in the cholinergic NBM and amygdala, we performed separate linear regressions for each group. The neurocognitive scores were considered as the dependent variable, whereas the volumetric measurements in bilateral NBMs and amygdalae, *APOE*-ε4, and interaction terms between the *APOE*-ε4 status and volume measurements in each ROI were considered as the independent variables, and the linear regressions were conducted using the following equation:

neurocognitive scores = NBM_L + NBM_R + amygdala_L + amygdala_R + *APOE*-ε4 + (*APOE*-ε4 × NBM_L) + (*APOE*-ε4 × NBM_R) + (*APOE*-ε4 × amygdala_L) + (*APOE*-ε4 × amygdala_R) + age + sex + edu + TIV

where NBM_L and NBM_R represent the left and right NBMs, respectively, and amygdala_L and amygdala_R represent the left and right amygdalae, respectively. Age, sex, edu, and TIV were also added as independent variables. The interaction terms between *APOE*-ε4 and VBM measurements were used to test the hypothesis that the relationship between neurocognitive performance and volumetric changes in the NBM and amygdala differs between *APOE*-ε4 carriers and non-carriers.

To investigate the effect of different levels of cognitive impairment on modulating the relationship between cognitive performance and volumetric changes in the cholinergic NBM and amygdala areas, we performed similar linear regressions for each disease group. The neurocognitive scores were considered as the dependent variable, whereas the volume measurements in bilateral NBMs and amygdalae, group (disease vs. normal control groups), and interaction terms between the group effect and volume measurements in each ROI were treated as the independent variables. Age, sex, edu, and TIV were added as the independent variables, and the linear regressions were conducted using the following expression:

neurocognitive scores = NBM_L + NBM_R + amygdala_L + amygdala_R + group + (group × NBM_L) + (group × NBM_R) + (group × amygdala_L) + (group × amygdala_R) + age + sex + edu + TIV

The interaction term between group and VBM measurements was used to test the hypothesis that the relationship between neurocognitive performance and volumetric changes in the NBM and the amygdala is different between the disease and CN groups.

All the statistical results are reported at the *P* < 0.05 significance level.

### Machine Learning Analysis

To test brain atrophy along with the neurocognitive features for classifying AD and CN patients, we implemented a machine learning (ML) algorithm using a non-linear support vector machine (SVM) with a radial basis function kernel (25, 26). SVM classifies a given set of data into two classes by constructing a hyperplane that maximizes the margin between classes (10). The 10-fold cross-validation was used to obtain the optimal parameters of gamma and overfitting constant *C* with the best accuracy (27–29). The data were randomly split into training and test data sets with a training/test ratio of 7:3. We computed specificity, sensitivity, and accuracy. For the classification, 15 features of interest were included in the analysis: the bilateral volume of CH123, CH4, and amygdala; TIV; *APOE*-ε4 status; demographic data including sex, age, and number of years of formal education; MEM; EF; LAN; and VS. The data were converted to *z*-scores. The area under the receiver-operating characteristic curve and the accuracy classification rates were used to evaluate classifier performance. We further evaluated the importance of the given features of neurocognitive and volumetric measurements for prediction results. In the SVM classification, we omitted the features of *APOE*-ε4 and neurocognitive or volumetric measurements and compared the performance with the whole feature set. The feature can be considered important if the resulting accuracy score decreased when the feature was omitted in the classification process (30).

## Results

### Demographics and Neurocognitive Measurements

Tables 1, 2 list the demographic and statistics of global composite scores for all groups. No significant difference was observed in sex, years of education, and TIV among all subject groups. We detected a considerably younger age of EMCI than that of the CN and AD groups. Thus, age was included as a covariate in all subsequent analyses. Two-way ANCOVA for neurocognitive measurements revealed the significant effect of group in MEM (*F* = 211.61, *P* < 0.0005), EF (*F* = 70.87, *P* < 0.0005), LAN (*F* = 779.06, *P* < 0.0005), and VS (*F* = 19.86, *P* < 0.0005). We found a significant *APOE*-ε4 effect on MEM (*F* = 11.40, *P* = 0.001). For MEM, *post-hoc* tests revealed that the MEM scores of EMCI, LMCI, and AD subjects were lower than those of CN subjects (*P* < 0.0005). For EF, *post-hoc* tests revealed that the EF scores of LMCI and AD subjects were lower than those of CN subjects (*P* < 0.0005). For LAN, *post-hoc* tests revealed that the LAN scores of EMCI, LMCI, and AD subjects were lower than those of CN subjects (*P* < 0.0005). For VS, VS scores of LMCI, and AD subjects were lower than those of CN subjects (*P* < 0.0005). *APOE*-ε4 carriers in LMCI showed worse MEM (*P* < 0.0005) and LAN (*P* = 0.045) performance than non-carriers. For EF, *APOE*-ε4 carriers in EMCI showed worse EF performance than non-carriers (*P* = 0.033). There was no interaction effect of *APOE*-ε4 × group on any of the neurocognitive scores.

**Table 1:** Demographic characteristics of five different subject groups.

		**CN (*n* = 167)**	**SMC (*n* = 96)**	**EMCI (*n* = 146)**	**LMCI (*n* = 138)**	**AD (*n* = 121)**	***F* or χ^**2**^ (*P*-value)**
Age	Mean ± SD	72.47 ± 6.18	72.02 ± 5.52	70.86 ± 6.92	71.87 ± 7.46	72.41 ± 8.10	0.002^*^
Sex	M/F	79/88	42/54	83/63	72/66	69/52	0.16
Education (years)	Mean ± SD	16.63 ± 2.48	16.71 ± 2.59	16.36 ± 2.61	16.39 ± 2.62	15.96 ± 2.51	0.17
TIV	Mean ± SD	1379.94 ± 133.93	1398.95 ± 137.32	1407.60 ± 137.56	1409.06 ± 147.90	1400.75 ± 156.06	0.38

*Group differences in age and education were assessed with a one-way analysis of variance (ANOVA) or χ^2^-test for categorical variables. CN, cognitively normal; SMC, significant memory concern; EMCI, early mild cognitive impairment; LMCI, late mild cognitive impairment; AD, Alzheimer's disease*.

* *P <0.05. Values that are statistically significant will be printed in bold*.

**Table 2:** Comparison between *APOE*-ε4 allele carriers and non-carriers in cognitive performances for each subject group.

**Group**	**Cognitive performances**	***APOE*-ε4 status**	**Mean (std)**	***P*-value**
CN	MEM	*APOE*-ε4 (–)	1.102 (0.603)	0.157
		*APOE*-ε4 (+)	1.012 (0.543)	
	EF	*APOE*-ε4 (–)	0.928 (0.801)	0.613
		*APOE*-ε4 (+)	0.867 (0.756)	
	LAN	*APOE*-ε4 (–)	0.876 (0.741)	0.562
		*APOE*-ε4 (+)	0.942 (0.597)	
	VS	*APOE*-ε4 (–)	0.289 (0.592)	0.585
		*APOE*-ε4 (+)	0.194 (0.515)	
SMC	MEM	*APOE*-ε4 (–)	1.092 (0.554)	0.498
		*APOE*-ε4 (+)	1.085 (0.615)	
	EF	*APOE*-ε4 (–)	0.778 (0.831)	0.893
		*APOE*-ε4 (+)	0.841 (0.879)	
	LAN	*APOE*-ε4 (–)	0.716 (0.661)	0.980
		*APOE*-ε4 (+)	0.802 (0.739)	
	VS	*APOE*-ε4 (–)	0.182 (0.600)	0.744
		*APOE*-ε4 (+)	0.253 (0.628)	
EMCI	MEM	*APOE*-ε4 (–)	0.670 (0.630)	0.091
		*APOE*-ε4 (+)	0.495 (0.579)	
	EF	*APOE*-ε4 (–)	0.693 (0.811)	0.033^*^
		*APOE*-ε4 (+)	0.411 (0.853)	
	LAN	*APOE*-ε4 (–)	0.593 (0.753)	0.121
		*APOE*-ε4 (+)	0.393 (0.751)	
	VS	*APOE*-ε4 (–)	0.113 (0.780)	0.310
		*APOE*-ε4 (+)	−0.031 (0.694)	
LMCI	MEM	*APOE*-ε4 (–)	0.180 (0.636)	<0.0005^*^
		*APOE*-ε4 (+)	−0.133 (0.605)	
	EF	*APOE*-ε4 (–)	0.263 (0.936)	0.284
		*APOE*-ε4 (+)	0.131 (0.831)	
	LAN	*APOE*-ε4 (–)	0.333 (0.766)	0.045^*^
		*APOE*-ε4 (+)	0.081 (0.805)	
	VS	*APOE*-ε4 (–)	−0.177 (0.757)	0.667
		*APOE*-ε4 (+)	−0.142 (0.739)	
AD	MEM	*APOE*-ε4 (–)	−0.872 (0.484)	0.532
		*APOE*-ε4 (+)	−0.885 (0.599)	
	EF	*APOE*-ε4 (–)	−0.882 (0.905)	0.572
		*APOE*-ε4 (+)	−0.771 (0.917)	
	LAN	*APOE*-ε4 (–)	−0.904 (0.814)	0.170
		*APOE*-ε4 (+)	−0.695 (1.018)	
	VS	*APOE*-ε4 (–)	−0.574 (0.720)	0.625
		*APOE*-ε4 (+)	−0.525 (1.027)	

*APOE-ε4 (+), APOE-ε4 carriers; APOE-ε4 (–), non-APOE-ε4 carriers; CN, cognitively normal; SMC, significant memory concern; EMCI, early mild cognitive impairment; LMCI, late mild cognitive impairment; AD, Alzheimer's disease; MEM, memory function; EF, executive function; LAN, language function; VS, visuospatial function*.

* *P <0.05. Values that are statistically significant will be printed in bold*.

### VBM Group Comparison Through ANCOVA

**Figure 1:**
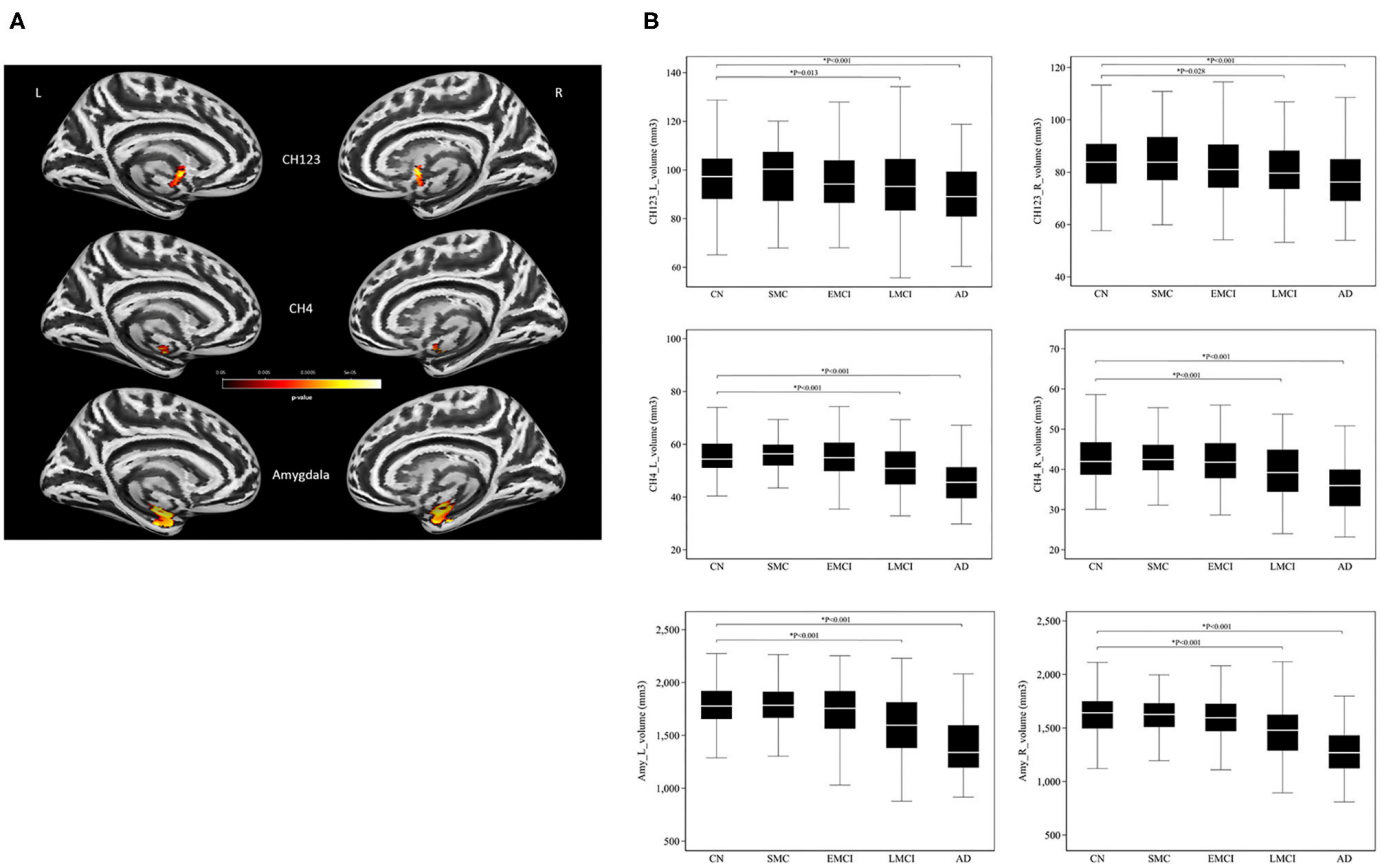
**(A)** The main effect of group differences on amygdala and basal forebrain subregion volume through voxel-based morphometry 5 × 2 ANCOVA (five diagnostic groups × *APOE*-ε4 carrier status) analysis. Significant atrophy of gray matter volume is presented on the inflated cortical surfaces (*P* < 0.05 family-wise error corrected). **(B)** Signficant atrophy in both basal forebrain and amygdala volume (in cubic millimeters) from CN to AD. *P < 0.05. CN, cognitively normal; SMC, significant memory concern; EMCI, early cognitive impairment; LMCI, late cognitive impairment; AD, Alzheimer's disease.

A significant main effect of the diagnostic group was observed in the amygdala (left: *P* = 1.52 × 10^−6^; right: *P* = 4.70 × 10^−6^) and the basal forebrain subregions (left Ch123: *P* = 0.002; right Ch123: *P* = 0.003; left NBM: *P* = 8.01 × 10^−6^; right NBM: *P* = 8.94 × 10^−5^; Figure 1A and Supplementary Table S2). The most pronounced atrophies occurred bilaterally in the amygdala and NBM. *Post-hoc* tests revealed considerably higher brain atrophy in the NBM–amygdala pathway in LMCI and AD subjects than in CN subjects (Figure 1B).

**Figure 2:**
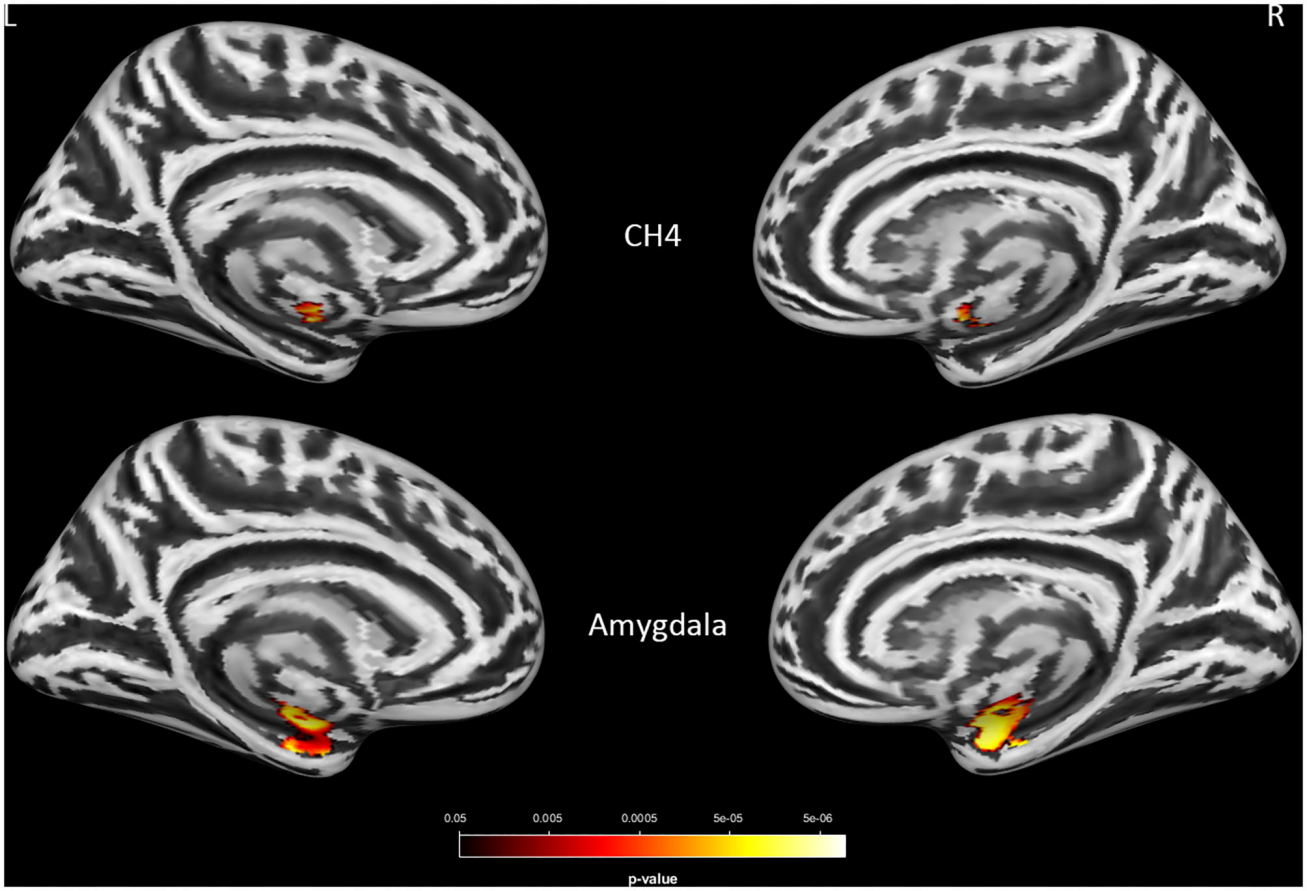
The main effect of *APOE*-ε4 on amygdala and basal forebrain subregion volume through voxel-based morphometry 5 × 2 ANCOVA (five diagnostic groups × *APOE*-ε4 carrier status) analysis. Significant atrophy of gray matter volume is presented on the inflated cortical surfaces (*P* < 0.05 family-wise error corrected).

Consistent with the finding obtained for the group effect, the significant main effect of *APOE*-ε4 was also observed bilaterally in the amygdala (left: *P* = 0.0004; right: *P* = 0.0005) and the NBM (left: *P* = 0.008; right: *P* = 0.023; Figure 2 and Supplementary Table S3) but not in the Ch123 (*P* > 0.05). Table 3 displays a comparison of *APOE*-ε4 carriers and non-carriers in each group. A *post-hoc* analysis indicated that *APOE*-ε4 carriers in the LMCI and AD groups had considerably reduced volumes of the NBM and amygdala than non-carriers.

**Table 3:** Volumetric comparison between *APOE*-ε4 allele carriers (+) and non-carriers (–).

		**CN**	**SMC**	**EMCI**	**LMCI**	**AD**
CH123 R	*APOE*-ε4+	83.18 ± 10.71	85.46 ± 9.75	83.57 ± 12.10	80.60 ± 13.12	76.15 ± 11.00
	*APOE*-ε4–	84.29 ± 12.22	84.51 ± 11.85	80.80 ± 11.61	80.17 ± 10.69	78.59 ± 11.38
	*P*-value	0.581	0.696	0.161	0.838	0.278
CH123 L	*APOE*-ε4+	97.20 ± 13.80	98.05 ± 13.16	97.37 ± 14.56	92.95 ± 16.05	88.72 ± 12.26
	*APOE*-ε4–	98.32 ± 15.21	98.20 ± 13.66	94.06 ± 13.79	93.11 ± 13.54	91.58 ± 14.16
	*P*-value	0.661	0.959	0.160	0.950	0.273
NBM R	*APOE*-ε4+	42.51 ± 6.09	43.42 ± 5.29	41.81 ± 5.60	37.80 ± 6.32	34.94 ± 6.32
	*APOE*-ε4–	42.92 ± 5.97	42.90 ± 6.36	41.93 ± 6.28	41.69 ± 6.34	37.59 ± 6.06
	*P*-value	0.689	0.692	0.902	0.001^*^	0.038^*^
NBM L	*APOE*-ε4+	56.05 ± 7.88	56.97 ± 5.51	54.92 ± 7.50	49.04 ± 8.39	44.93 ± 8.13
	*APOE*-ε4–	56.35 ± 7.96	56.88 ± 8.70	55.17 ± 8.82	53.81 ± 7.22	48.19 ± 8.34
	*P*-value	0.824	0.954	0.860	0.001^*^	0.051
Amy R	*APOE*-ε4+	1617.03 ± 164.02	1627.80 ± 134.35	1593.24 ± 227.54	1388.45 ± 254.34	1234.49 ± 218.26
	*APOE*-ε4–	1639.51 ± 197.94	1623.25 ± 217.06	1570.07 ± 228.49	1546.16 ± 242.24	1382.91 ± 258.71
	*P*-value	0.487	0.914	0.542	<0.0005^*^	0.002^*^
Amy L	*APOE*-ε4+	1783.82 ± 223.15	1804.56 ± 158.52	1745.64 ± 253.36	1501.07 ± 306.62	1339.72 ± 257.36
	*APOE*-ε4–	1800.93 ± 220.85	1801.75 ± 239.93	1709.38 ± 270.49	1705.07 ± 242.15	1485.02 ± 291.34
	*P*-value	0.652	0.952	0.407	<0.0005^*^	0.008^*^

*Data are presented in unit of cubic millimeter of mean volume ± standard deviation adjusted for age, sex, edu, and total intracranial volume*.

* *P <0.05. NBM, nucleus basalis of Meynert; Amy, amygdala; CN, cognitively normal; SMC, significant memory concern; EMCI, early mild cognitive impairment; LMCI, late mild cognitive impairment; AD, Alzheimer's disease; MEM, memory function; EF, executive function; LAN, language function; VS, visuospatial function. Values that are statistically significant will be printed in bold*.

**Figure 3:**
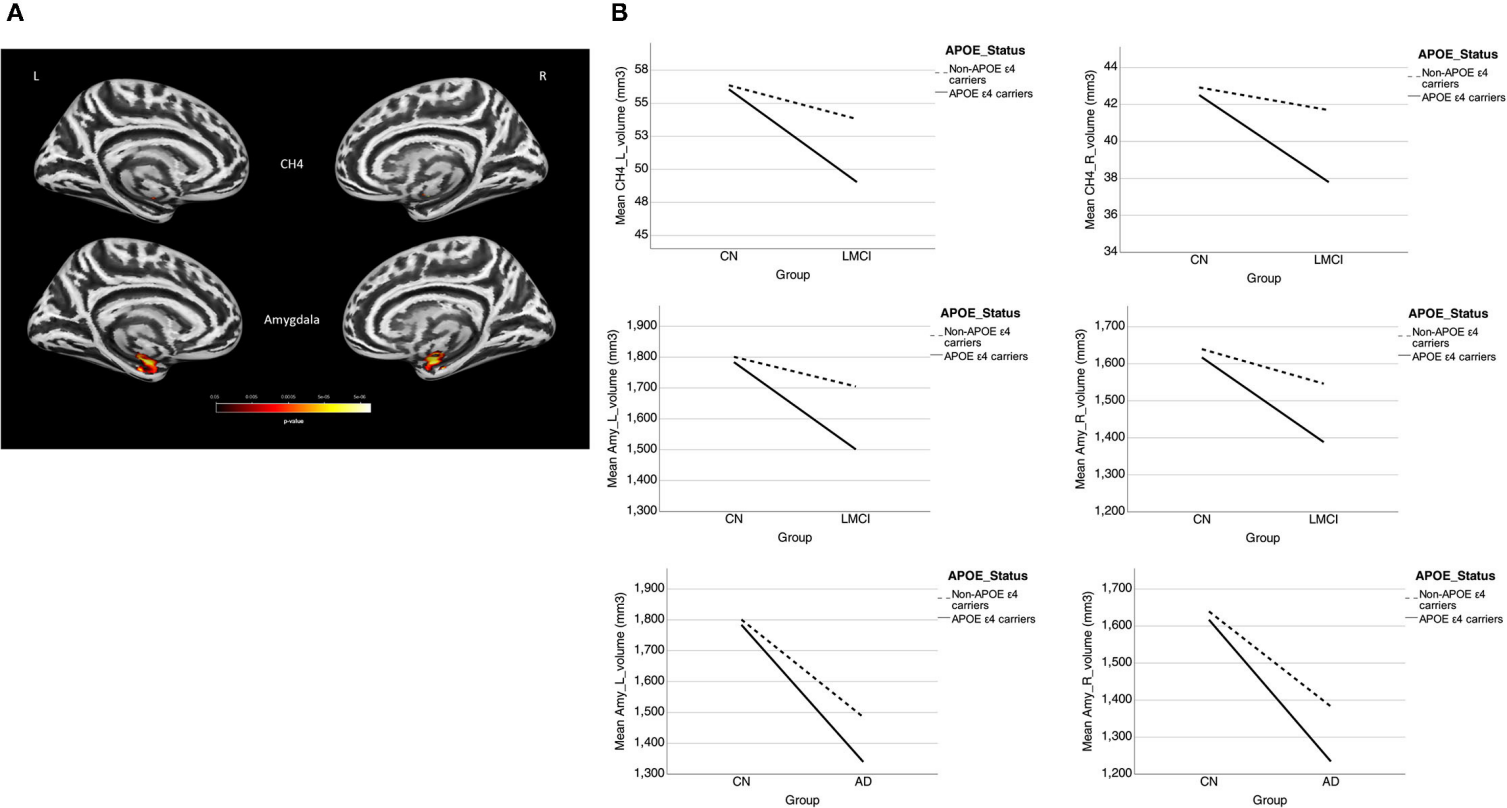
**(A)** Interaction effect of *APOE*-ε4 × group on amygdala and basal forebrain subregion volume through voxel-based morphometry 5 × 2 ANCOVA (five diagnostic groups × *APOE*-ε4 carrier status) analysis. Significant atrophy of gray matter volume is presented on the inflated cortical surfaces (*P* < 0.05 family-wise error corrected). **(B)** The significant interaction effect of *APOE*-ε4 × group on bilateral amygdala and NBM subregion mean volume (in cubic millimeter) in patients with LMCI and AD as compared with CN subjects. *APOE*-ε4 carriers exhibited more volume reduction than non-carriers in the LMCI and AD groups, while there is no significant difference between *APOE*-ε4 carriers and non-carriers in the CN group. CN, cognitively normal; LCMI, late cognitive impairment; AD, Alzheimer's disease.

A significant interaction effect of *APOE*-ε4 × group was observed bilaterally in the NBM (*P* < 0.0005) and amygdala (*P* < 0.0005) for LMCI vs. CN subjects and in the NBM (*P* = 0.007 and 0.004 for left and right NBM, respectively) for AD vs. CN subjects. *APOE*-ε4 carriers exhibited more volume reduction than non-carriers in the LMCI and AD groups, while there is no significant difference between carriers and non-carriers in the CN group (Figure 3A). This finding suggests a significant difference in how *APOE*-ε4 affects the amygdala and NBM volume loss for the LMCI and AD groups compared with the CN group (Figure 3B and Supplementary Table S4). Again, no significant interaction effect of *APOE*-ε4 with the group was observed for Ch123.

Supplementary Tables S5A–G shows the ANCOVA results of group comparison with different allelic numbers in each diagnostic group. We found a significant group effect in MEM and atrophy in the bilateral NBM and amygdala for LMCI. We also found a significant group effect in the bilateral amygdala for AD. However, no significant group difference was found between *APOE*-ε4-homozygous and ε4-heterozygous subject groups in the results of *post-hoc* tests (Supplementary Tables S5E,G).

### Machine Learning Analysis

**Figure 4:**
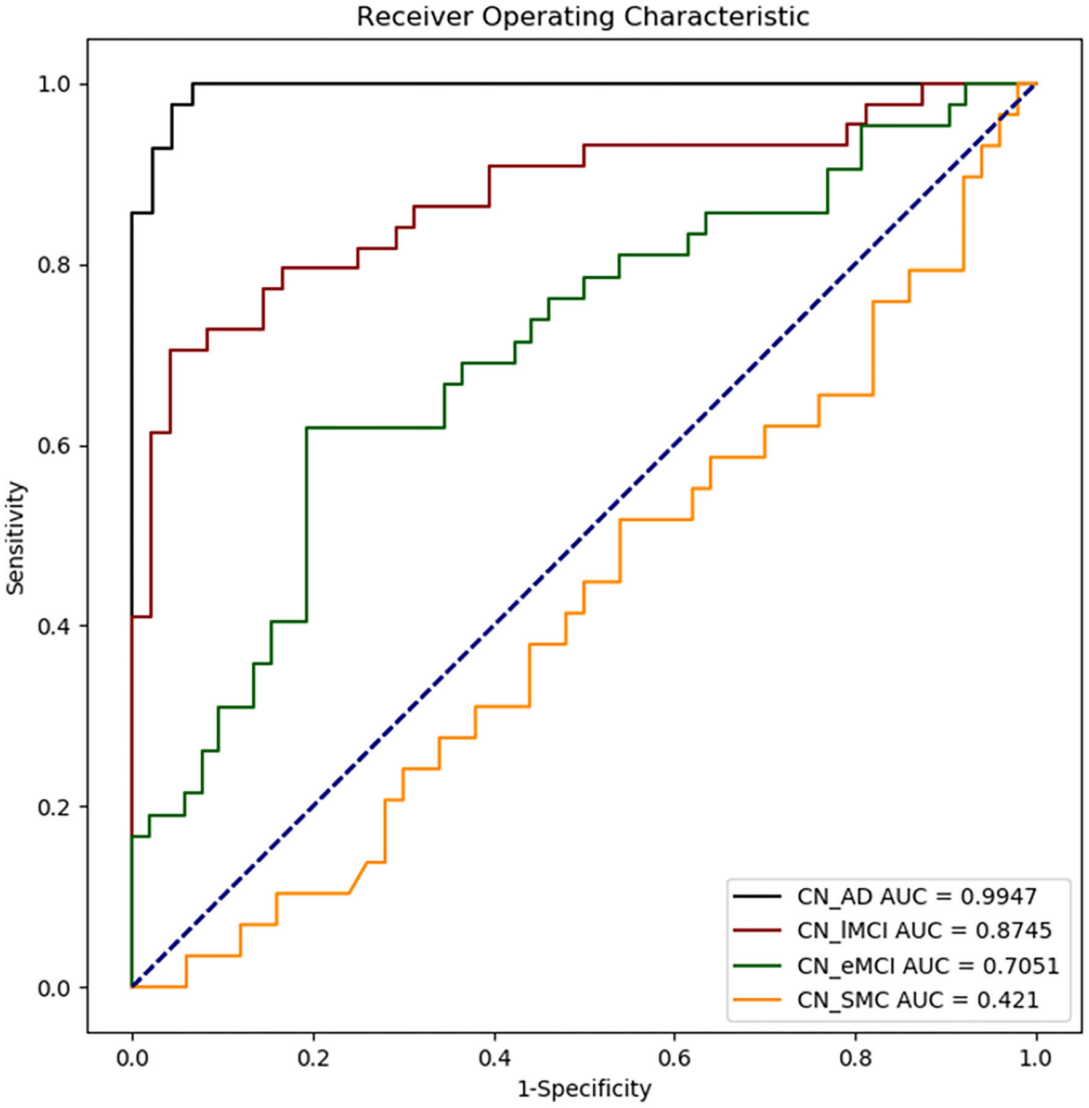
Receiver operating characteristic (ROC) curves obtained for each disease group as compared with CN. CN_AD, comparison between CN and AD; CN_lMCI, comparison between CN and LCMI; CN_eMCI, comparison between CN and ECMI; CN_SMC, comparison between CN and SMC; CN, cognitively normal; SMC, significant memory concern; EMCI, early cognitive impairment; LMCI, late cognitive impairment; AD, Alzheimer's disease.

The results of ML classification are illustrated in Figure 4 and Table 4. The combination of features yielded classification accuracy rates of 0.468, 0.648, 0.804, and 0.966 for SMC, EMCI, LMCI, and AD, respectively. Supplementary Table S6 shows the results of classification accuracy for all features (All) and excluding *APOE*-ε4 status (All-*APOE*-ε4), neurocognitive performance (All-neurocognitive performance), or VBM (All-VBM) measurements at a time from the overall model. The resulting accuracy score does not significantly decrease without the *APOE*-ε4 feature. However, the classification accuracy decreases when the neurocognitive or VBM measurements were removed. The classification accuracy in AD vs. CN decreases the most when VBM measurements were removed.

**Table 4:** Machine learning classification outcomes for each diseased group as compared with CN group.

	**SMC-CN**	**EMCI-CN**	**LMCI-CN**	**AD-CN**
AUC	0.421	0.705	0.875	0.995
Acc	0.468	0.648	0.804	0.966
Spec	0.520	0.635	0.812	0.956
Sens	0.379	0.667	0.795	0.976

*AUC, area under the ROC; Acc, accuracy; Spec, specificity; Sens, sensitivity; CN, cognitively normal; SMC, significant memory concern; EMCI, early mild cognitive impairment; LMCI, late mild cognitive impairment; AD, Alzheimer's disease*.

### Structural Relationships Between the Amygdala and NBM

**Figure 5:**
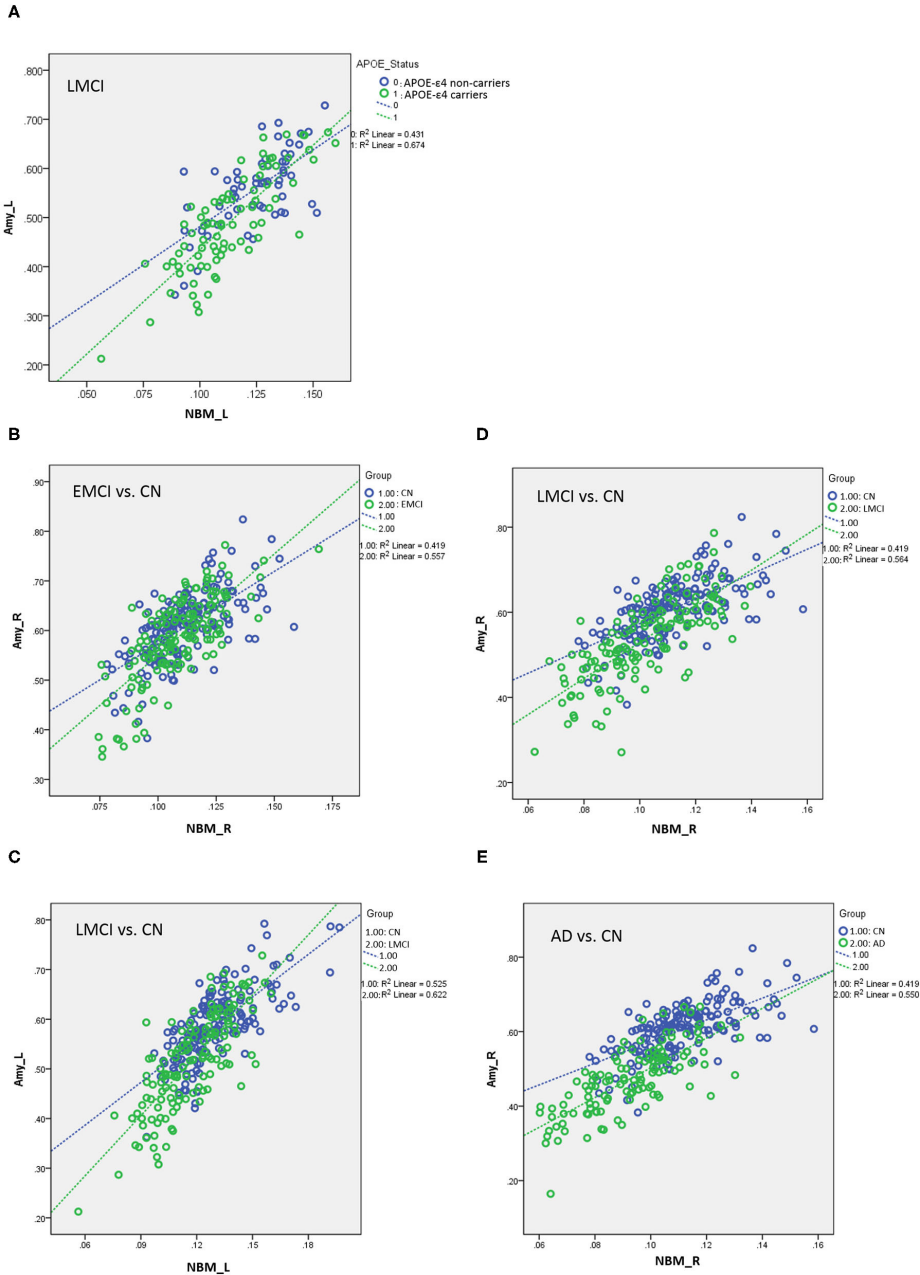
**(A)** Plots of the associations between the NBM and the amygdala for *APOE*-ε4 carriers and non-carriers in LMCI. Different slopes of the regression lines indicated the interaction effect of *APOE*-ε4 by the NBM [using the following regression model: amygdala = NBM + *APOE*-ε4 + (NBM × *APOE*-ε4) + age + sex + edu + TIV] in the volumetric changes of the amygdala. *APOE*-ε4 carriers showed a stronger structural correlation between the NBM and the amygdala than non-carriers. Plots of the associations between the NBM and the amygdala for **(B)** EMCI vs. CN, **(C,D)** LMCI vs. CN, and **(E)** AD vs. CN using the following regression model: amygdala = NBM + group + (NBM × group) + age + sex + edu + TIV. Different slopes of the regression lines indicated the interaction effect of group by the NBM in the volumetric changes of the amygdala. Disease groups showed a stronger structural correlation between the NBM and the amygdala than CN subjects. NBM, nucleus basalis of Meynert; Amy, amygdala; CN, cognitively normal; EMCI, early mild cognitive impairment; LMCI, late cognitive impairment; AD, Alzheimer's disease; _R, right side; _L, left side. Data are presented in normalized volume.

Table 5 presents the results of the *APOE*-ε4 effect on the structural associations for different subject groups. All groups exhibited a significant positive correlation of volume between the bilateral NBM and amygdala volumes, with the exception of the SMC group, which exhibited a correlation only in the right side. The *APOE*-ε4 status was associated with lower amygdala volumes in the LMCI group (*APOE*-ε4 effect in LMCI, beta coefficient = −0.162, *P* = 0.017). The LMCI group also revealed a significant interaction effect between *APOE*-ε4 and the left NBM in the association with amygdala volume, which suggested a significant effect of *APOE*-ε4 on the correlation between volumes in the left NBM and amygdala. *APOE*-ε4 carriers exhibited a significantly stronger structural correlation between volumes in the left NBM and amygdala than non-carriers in the LMCI group (Figure 5A).

**Table 5:** Statistical results with *APOE*-ε4 effect on the relation between the amygdala and NBM using the following regression model: amygdala = NBM + *APOE*-ε4 + (NBM × *APOE*-ε4) + age + sex + edu + TIV.

	**CN**		**SMC**		**EMCI**		**LMCI**		**AD**	
	**Amy_L**	**Amy_R**	**Amy_L**	**Amy_R**	**Amy_L**	**Amy_R**	**Amy_L**	**Amy_R**	**Amy_L**	**Amy_R**
*F* (*P*-value)	81.45 (<0.0005)	59.58 (<0.0005)	57.36 (<0.0005)	51.45 (<0.0005)	70.19 (<0.0005)	101.70 (<0.0005)	70.89 (<0.0005)	57.64 (<0.0005)	47.71 (<0.0005)	57.54 (<0.0005)
NBM, β (*P*-value)	2.174 (<0.0005)	1.973 (<0.0005)	NS	1.96 (<0.0005)	2.88 (<0.0005)	3.77 (<0.0005)	2.90 (<0.0005)	3.83 (<0.0005)	3.01 (<0.0005)	3.92 (<0.0005)
*APOE*-ε4, β (*P*-value)	NS	NS	NS	NS	NS	NS	−0.162 (0.017)	NS	NS	NS
*APOE*-ε4 × NBM, β (*P*-value)	NS	NS	NS	NS	NS	NS	1.098 (0.04^*^)	NS	NS	NS
Age, β (*P*-value)	−0.001 (0.036)	−0.002 (0.009)	−0.005 (<0.0005)	−0.003 (<0.0005)	−0.002 (0.003)	NS	−0.003 (<0.0005)	−0.003 (<0.0005)	−0.003 (<0.0005)	NS
Sex, β (*P*-value)	NS	NS	NS	NS	0.038 (<0.0005)	NS	NS	0.025 (0.020)	NS	NS
Edu, β (*P*-value)	NS	NS	NS	NS	NS	NS	NS	NS	NS	NS
TIV, β (*P*-value)	<0.0005 (<0.0005)	<0.0005 (<0.0005)	<0.0005 (<0.0005)	<0.0005 (<0.0005)	NS	<0.0005 (0.001)	NS	NS	<0.0005 (0.011)	NS

*Amy_L, left amygdala; Amy_R, right amygdala; NS, not significant; F, F-test value of the regression model fit; NBM, nucleus basalis of Meynert; TIV, total intracranial volume; Edu, educational level; CN, cognitively normal; SMC, significant memory concern; EMCI, early mild cognitive impairment; LMCI, late mild cognitive impairment; AD, Alzheimer's disease*.

* *Significant interaction term of APOE-ε4 × NBM indicating that the relationship between the volumetric changes in the NBM and the amygdala is different in APOE-ε4 carriers and non-carriers. Values that are statistically significant will be printed in bold*.

Table 6 presents the results of structural regression analysis of the group effect. As compared with CN subjects, we observed the group effect of bilateral amygdala volumetric changes in EMCI, LMCI, and AD subjects. EMCI, LMCI, and AD patients exhibited greater brain atrophy in the amygdala than CN subjects. Regression analyses revealed significant interactions between the group and NBM volume in EMCI (right side: β = 1.792, *P* < 0.0005), LMCI (left side: β = 0.434, *P* < 0.0005 and right side: β = 0.533, *P* < 0.0005), and AD (right side: β = 0.987, *P* = 0.014) patients (Figures 5B–E). The direction of these interaction effects was stronger in the EMCI, LMCI, and AD groups than in the CN group, which indicated that these disease groups exhibited stronger correlations between NBM and amygdala volumetric changes than the CN group.

**Table 6:** The effect of group on the relation between the amygdala and NBM using the following regression model: amygdala = NBM + group + (NBM × group) + age + sex + edu + TIV.

	**SMC vs. CN**		**EMCI vs. CN**		**LMCI vs. CN**		**AD vs. CN**	
	**Amy_L**	**Amy_R**	**Amy_L**	**Amy_R**	**Amy_L**	**Amy_R**	**Amy_L**	**Amy_R**
*F* (*P*-value)	84.83 (<0.0005)	106.58 (<0.0005)	94.39 (<0.0005)	99.483 (<0.0005)	122.54 (<0.0005)	100.382 (<0.0005)	224.379 (<0.0005)	151.150 (<0.0005)
NBM, β (*P*-value)	1.583 (<0.0005)	2.003 (<0.0005)	2.458 (<0.0005)	NS	1.923 (<0.0005)	1.648 (<0.0005)	2.517 (<0.0005)	1.259 (0.044)
Group, β (*P*-value)	NS	NS	−0.026 (<0.0005)	−0.218 (<0.0005)	−0.066 (<0.0005)	−0.073 (<0.0005)	−0.077 (<0.0005)	−0.185 (<0.0005)
Group × NBM, β (*P*-value)	NS	NS	NS	1.792 (<0.0005^*^)	0.434 (<0.0005^*^)	0.533 (<0.0005^*^)	NS	0.987 (0.014^*^)
Age, β (*P*-value)	−0.002 (<0.0005)	−0.002 (<0.0005)	−0.002 (<0.0005)	−0.001 (0.003)	−0.002 (<0.0005)	−0.002 (<0.0005)	−0.002 (<0.0005)	−0.001 (0.003)
Sex, β (*P*-value)	0.017 (0.021)	NS	0.022 (0.003)	NS	NS	NS	NS	NS
Edu, β (*P*-value)	NS	NS	NS	NS	NS	NS	NS	NS
TIV, β (*P*-value)	<0.0005 (<0.0005)	<0.0005 (<0.0005)	<0.0005 (<0.0005)	<0.0005 (<0.0005)	<0.0005 (<0.0005)	<0.0005 (<0.0005)	<0.0005 (<0.0005)	<0.0005 (<0.0005)

*Amy_L, left amygdala; Amy_R, right amygdala; NS, not significant; F, F-test value of the regression model fit; CN, cognitively normal; SMC, significant memory concern; EMCI, early mild cognitive impairment; LMCI, late mild cognitive impairment; AD, Alzheimer's disease*.

* *Significant interaction term of group × NBM indicating that the relationship between the volumetric changes in the NBM and the amygdala is different between disease subjects and cognitively normal subjects. Values that are statistically significant will be printed in bold*.

### Correlation Between VBM and Neurocognitive Measurements

**Figure 6:**
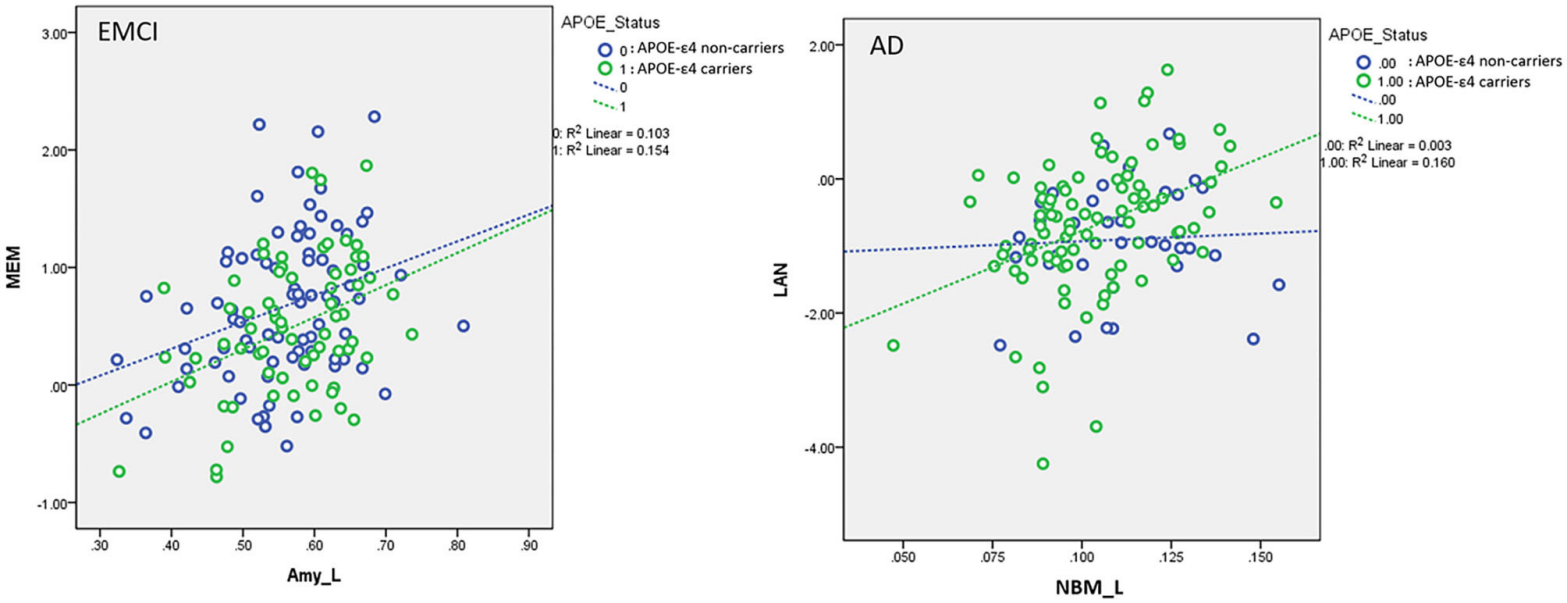
Plots of the associations between the left amygdala and memory function in EMCI **(Left)** and between the left NBM and language function in AD **(Right)** using the following regression model: neurocognitive scores = NBM_L + NBM_R + amygdala_L + amygdala_R + *APOE*-ε4 + (*APOE*-ε4 × NBM_L) + (*APOE*-ε4 × NBM_R) + (*APOE*-ε4 × amygdala_L) + (*APOE*-ε4 × amygdala_R) + age + sex + edu + TIV. Different slopes of the regression lines indicated the interaction effects of *APOE*-ε4 × left amygdala in MEM for EMCI and *APOE*-ε4 × left NBM in LAN for AD subjects, respectively. *APOE*-ε4 carriers exhibited a stronger correlation between brain morphology and neurocognitive performance than non-carriers for EMCI and AD. NBM_L, left nucleus basalis of Meynert; Amy_L, left amygdala; EMCI, early mild cognitive impairment; AD, Alzheimer's disease; MEM, memory function; LAN, language function. Data are presented in normalized volume.

For the association between cholinergic volumetric changes and cognitive performance (Supplementary Table S7), we observed significant effects of the left amygdala on the MEM (EMCI, LMCI, and AD); left NBM (CN), left amygdala (SMC and AD), and right amygdala (LMCI) on EF; left NBM (EMCI) and left amygdala (SMC, LMCI, and AD) on LAN; and left NBM (CN and EMCI) on VS. The significant interaction effect between *APOE*-ε4 and VBM measurements implies that *APOE*-ε4 plays a crucial role in modulating the association between cerebral morphology and neurocognitive performance. For MEM, we observed a significant interaction effect between the *APOE*-ε4 status and the left amygdala in the EMCI group (β = 0.35, *P* = 0.015). *APOE*-ε4 carriers showed a stronger correlation between the left amygdala and MEM than non-carriers in the EMCI group (Figure 6). For LAN, we observed a significant interaction effect between the *APOE*-ε4 status and the left NBM in AD patients (β = 3.38, *P* = 0.03). *APOE*-ε4 carriers exhibited a stronger correlation between the left NBM and LAN than non-carriers in the AD group (Figure 6).

**Figure 7:**
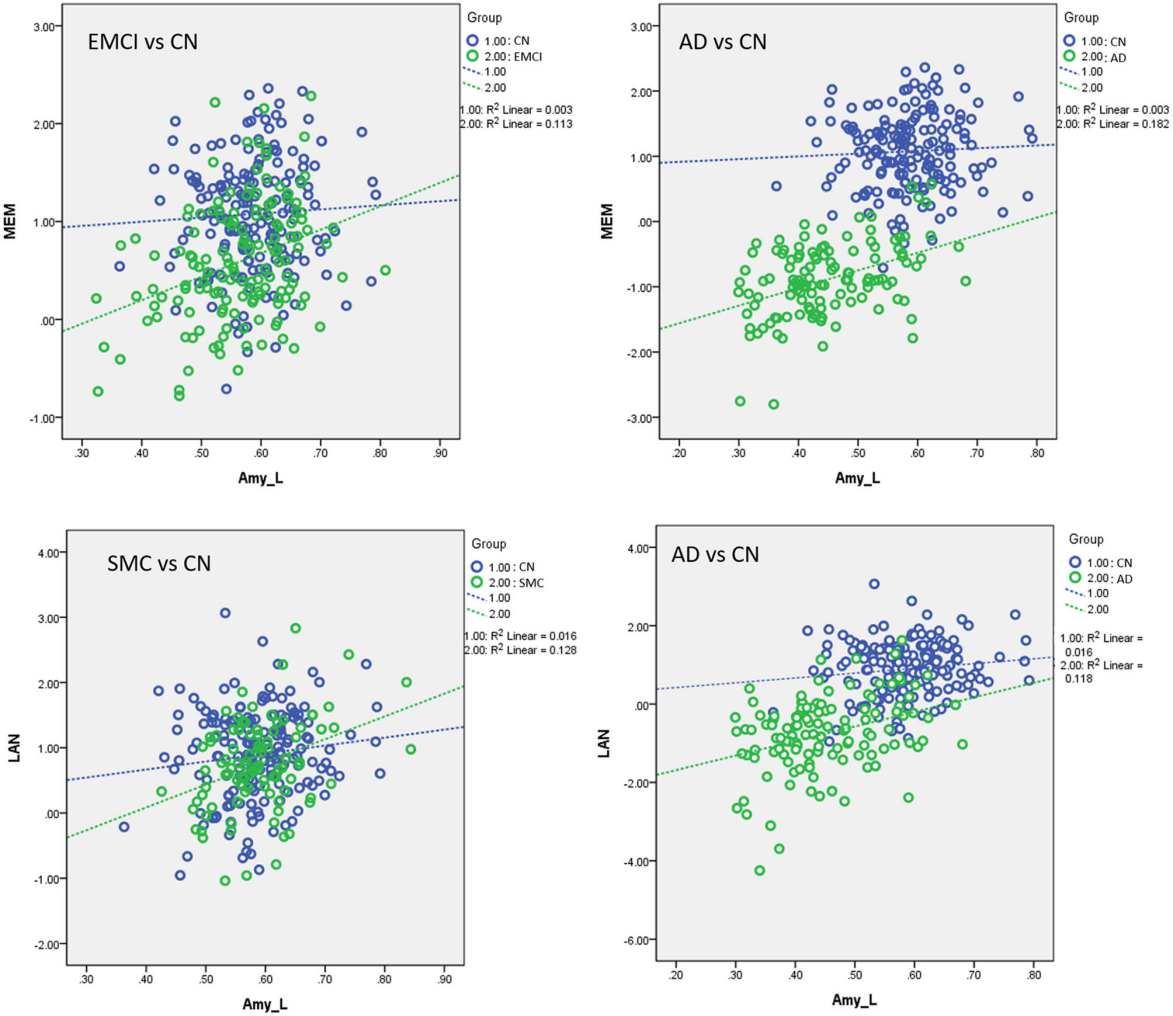
Plots of the associations between volumetric and neurocognitive measurements using the following regression model: neurocognitive scores = NBM_L + NBM_R + amygdala_L + amygdala_R + group + (group × NBM_L) + (group × NBM_R) + (group × amygdala_L) + (group × amygdala_R) + age + sex + edu + TIV. Different slopes of the regression lines indicated the interaction effect of group by brain morphology in neurocognitive performance. Disease groups showed stronger correlations between brain morphology and neurocognitive performance than CN subjects. Amy_L, left amygdala; MEM, memory function; LAN, language function; CN, cognitively normal; SMC, significant memory concern; EMCI, early mild cognitive impairment; AD, Alzheimer's disease. Data are presented in normalized volume.

Supplementary Table S8 displays the results of linear regression analyses with effects of group and interaction of group × VBM measurements in modulating the neurocognitive performance. For MEM, we found significant interaction effects of group by the left amygdala in EMCI vs. CN (β = 1.006, *P* < 0.0005) and AD vs. CN subjects (β = 1.49, *P* < 0.0005). EMCI and AD subjects exhibited stronger correlations between MEM and the amygdala than CN subjects. For LAN, a significant interaction effect of group and the amygdala was observed in SMC vs. CN (β = 1.08, *P* = 0.01) and AD vs. CN subjects (β = 3.20, *P* < 0.0005). SMC and AD patients exhibited stronger correlations between LAN and the amygdala than control subjects (Figure 7). No significant interaction effect of group × VBM measurements was observed in the association between brain volumetric changes and neurocognitive performance in EF and VS.

## Discussion

### Major Findings

To the best of our knowledge, no study has systematically assessed the effect of *APOE-*ε*4* on the associations with the cholinergic structural changes and neurocognitive performance for subjects with different levels of cognitive impairment. We evaluated the cerebral morphological changes of various groups and the effect of *APOE-*ε*4* on brain atrophy, structural association between the NBM and amygdala, and the association between volumetric changes and neurocognitive performance, focusing on the cholinergic brain regions. We found that the effect of the *APOE-*ε*4* allele on brain atrophy in the disease groups differs from that in the control group. For LMCI and AD groups, *APOE*-ε4 allele carriers exhibited higher brain atrophy than non-carriers in the cholinergic pathway (Figure 3B). No group difference was observed between *APOE*-ε4 allele carriers and non-carriers in the CN group. Structural association analyses revealed that *APOE*-ε4 allele carriers in the LMCI group exhibited a stronger correlation between the NBM and amygdala than non-carriers did (Figure 5A), and no difference was observed in the cholinergic structural association between the *APOE*-ε4 allele carriers and non-carriers in the CN group. Comparing the cholinergic structural association between the disease groups and the CN group revealed that EMC, LMCI, and AD subjects exhibited a stronger structural correlation in this pathway than CN did (Figures 5B–E). Regarding the correlation between volumetric and neurocognitive measurements, the *APOE*-ε4 allele carriers in the EMCI group exhibited a stronger correlation between the amygdala and MEM than non-carriers (Figure 6). The *APOE*-ε4 allele carriers in the AD group exhibited a stronger correlation between the NBM and LAN than non-carriers (Figure 6). For comparison of the correlation between volumetric and neurocognitive measurements between the disease and CN groups, EMCI and AD subjects exhibited a stronger correlation between the amygdala and MEM than CN subjects did (Figure 7). SMC and AD subjects exhibited a stronger correlation between the amygdala and LAN than CN subject did (Figure 7).

### Voxel-Based Morphometry in Two-Way ANCOVA

#### Main Effect of Group in Two-Way ANCOVA

We found the most pronounced atrophies bilaterally in the NBM and amygdala, although significant reductions were also evident in Ch123. A trend of gradually reducing basal forebrain and amygdala volume was observed from CN to AD over the different levels of cognitive impairment (Figure 1B), which reached statistical significance in both the LCMI and AD groups. The NBM and amygdala are anatomically and functionally connected as part of the anatomical segregation in the cholinergic projections, in which the amygdala is the distinct target of the cholinergic projections of the NBM subregion. The observed pattern of structural findings is consistent with the neurocognitive results that the LMCI and AD groups exhibited the most deterioration among these groups. No significant group difference was observed between SMC and CN in structural imaging and neurocognitive performance. For EMCI, however, a simple pair comparison with the CN group revealed that the deterioration of the neurocognitive function was more pronounced than that for CN, whereas the trend of reduced volume in structural measurements did not reach statistical significance as compared with the CN group. This finding may reflect the heterogeneity of the structural damage of the degenerative process that the GM loss may vary substantially in the early stage of preclinical AD cases (31).

We measured the NBM and amygdala volume for various disease groups as training features and classified the groups using the ML algorithm. We highlighted the features for classifying various disease and CN groups using the support vector regression algorithm and reported the specificity, sensitivity, and classification accuracy rates. The statistical results of group comparison were consistently confirmed using the ML algorithm, which revealed a gradual reduction of the prediction power in classifying the disease groups vs. the control group from AD to SMC, reflecting the severity of neuropathological changes at different levels of cognitive impairment. Regarding the importance of the given features for prediction results (Supplementary Table S6), *APOE*-ε4 feature does not seem to have a strong effect on the result of the group classification because the resulting accuracy score does not significantly decrease without the *APOE*-ε4 feature. This may be attributed to the small number of *APOE*-ε4 carriers in the control group. Both the neurocognitive and VBM measurements are considered to be relevant in the group classification results because the classification accuracy decreases when the neurocognitive or VBM measurements were removed. Discrimination of AD patients through VBM measurements was higher than results based on cognitive measures because the classification accuracy in AD vs. CN decreases the most when VBM measurements were removed.

#### The Main Effect of APOE-ε4 in Two-Way ANCOVA

We confirmed the *APOE*-ε4 effect on the brain atrophy in the cholinergic projections. In the LMCI and AD groups, *APOE*-ε4 carriers exhibited a lower NBM and amygdala volume than non-carriers, suggesting a considerable risk of the APOE genotype for cerebral morphology. This finding is consistent with the findings of group difference in the cognitive performance for LMCI patients that *APOE*-ε4 carriers showed a worse cognitive performance as compared with non-carriers. The trend of more reduced brain volume in the structural MRI for *APOE*-ε4 carriers remains the same in the AD stage. However, no significant difference was observed in neurocognitive performance between the *APOE*-ε4 carriers and non-carriers for patients with AD. Our finding is consistent with previous studies showing that *APOE*-ε4 carriers in AD did not have any group difference as compared with non-carriers in neurocognitive tests (32, 33). In AD, cognitive function may be affected considerably for the pathology status in this stage, and no difference was observed in neurocognitive performance between carriers and non-carriers. Alternatively, neuropsychological tests have been reported to be elusive with the limited sensitivity and specificity in detecting the direct effect of APOE on a cognitive phenotype (34, 35). Our finding may imply that structural MRI preserves the information of the *APOE*-ε4 effect on the brain structural deterioration, while there is no difference observed in neurocognitive performance between *APOE*-ε4 carriers and non-carriers for patients in the late stage of the AD spectrum. As mentioned in a previous report showing a higher hippocampal loss in the presence of *APOE*-ε4, the authors suggested that increased brain volume loss could be an indicator of Alzheimer's disease pathology and a potential marker for the efficacy of therapeutic interventions in Alzheimer's disease (36).

Early age-related basal forebrain neuronal loss in the cholinergic neuronal population may result in an imbalance of cholinergic control of microglia, allowing for an excess of pro-inflammatory-activated microglia. Neuroinflammation may occur in the hippocampus because the cholinergic inputs to the hippocampus are no longer present after the basal forebrain blockage. The results of the study conducted by Schmitz et al. (37) suggest that there is a correlation between the presence of basal forebrain degeneration and an increase in the magnitude of CSF-triggering receptor expressed on myeloid cells 2 (sTREM2) levels and increased levels of peripheral complement component 3 (C3) expression in the blood transcriptome. They suggested that a disruption in lipid metabolism could be the cause of cholinergic neuron damage in the aging brain and that this could be linked to an *APOE*-ε4 genetic background (37). In animal studies, researchers discovered that preclinical *APOE*-ε4 carriers had the highest levels of basal forebrain degeneration and C3 expression, even though CSF amyloid beta peptide (Aβ) and phosphorylated-tau (pTau) levels were equivalent (37). These findings corroborate prior research indicating that *APOE*-ε4 glia secretes less deposited cholesterol and fatty acids and deprives neurons of energy to construct, maintain, and repair synapses and axons (38–40). Cholinergic axon arbors have the additional drawback of increasing the burden on the cell for all of these processes, causing cells to age prematurely (41). Taken together, the findings suggest that AD neuroinflammation may be due in part to diminished input of cholinergic afferent basal forebrain neurons, which might also interrupt anti-inflammatory cholinergic signaling and drive further neurodegeneration.

By accounting for the effect of *APOE*-ε4 allelic number, the small number of *APOE*-ε4-homozygous (HO) subjects in each group (see Supplementary Tables S5A–G) raises a concern about the accuracy of the results, especially for groups in CN and SMC, which have a small number of HO subjects. No significant group difference was found between *APOE*-ε4-homozygous and ε4-heterozygous subject groups in the results of neurocognitive and volume measurements (Supplementary Tables S5E,G). This could be attributed to the smaller number of HO subjects in CN and SMC in our study.

#### Interaction Effect of APOE-ε4 × Group in Two-Way ANCOVA

As compared with the CN subjects, significantly different levels of brain atrophy in the cholinergic projections were found between *APOE*-ε4 carriers and non-carriers in the LMCI and AD groups. In the LMCI and AD groups, *APOE*-ε4 carriers showed significantly higher levels of volume reduction than non-carriers did, whereas no significant difference in volumetric changes was found between carriers and non-carriers in control subjects (Figure 3B). This finding is similar to a previous report of hippocampal atrophy analysis in LCMI and AD, in which *APOE*-ε4 carriers exhibited greater hippocampal atrophy than non-carriers, whereas no significant difference was found between *APOE*-ε4 genotypes in the subjects with normal cognition (42). These findings support the hypothesis that the *APOE*-ε4 genotype could modify the progress of brain atrophy over AD progression. *APOE*-ε4 may shift the hypothetical model of dynamic biomarkers of the AD's pathological cascade leftward during disease progression (42, 43). It has been suggested that the underlying pathophysiology may be attributed to the influence of the *APOE*-ε4 genotype on higher levels of Aβ deposition and higher degree and faster rate of neurodegeneration, thus resulting in more changes in the brain volume for the disease groups (42).

### Structural Association: Volumetric Changes in the NBM Associated With Atrophy in the Amygdala

#### APOE-ε4 Effect on the Structural Association

We found significant associations of volumetric changes between the NBM and amygdala bilaterally for CN, EMCI, LMCI, and AD and right side in SMC. Patients with LCMI exhibited a significant effect of *APOE*-ε4 on the volumetric changes for this cholinergic pathway. *APOE*-ε4 carriers in the LMCI exhibited stronger structural associations between the left NBM and amygdala than non-carriers did (Figure 5A). Regarding the progression of the disease pathogenesis, it has been reported that the degeneration of the NBM precedes and predicts entorhinal degeneration (44). The enhanced structural association of the volume reduction between the NBM and the amygdala in the cholinergic system for the *APOE*-ε4 carriers may imply that the increasing vulnerability caused by the *APOE*-ε4 effect could speed up the spread of the pathology across networks through the projection of cholinergic neurons in these patients (44). A previous report further suggested that the abnormal proteopathic pTau/Aβ accumulation may greatly exacerbate the neurodegeneration of the cholinergic basal forebrain system (44), in which *APOE*-ε4 has been related to amyloid-β and tau pathology in previous studies (42).

#### Group Difference of the Structural Association

In linear regression analyses, the significant finding of the interaction effect of the group by the NBM on the volumetric changes in the amygdala suggests that different groups showed different effects on the association between the volumetric changes in the NBM and amygdala. Patients with EMCI, LMCI, and AD showed significantly stronger associations between the volumetric changes in the NBM and amygdala than CN subjects did (Figures 5B–E). These findings are consistent with a previous report showing a stronger correlation of volumetric changes between the NBM and amygdala in EMCI compared with CN, which has a diagnostic value to differentiate with CN (7). We extended the previous report by showing a significant result in LMCI and AD. The enhanced association of the brain atrophy in the pathway between the NBM and amygdala can be speculated as the parallel disruption of the network of the NBM and its innervated region in the amygdala, likely revealing synaptic abnormality, as reported in a previous study showing increased levels of resting-state electroencephalogram functional connectivity in MCI subjects (45–47). The authors suggested that the cerebral cortex is characterized by a profound reorganization of intra- and inter-hemispheric metabolic connections during the progression of AD, leading to increased local interactions at the expense of long-range connections (47). Alternatively, this finding could be related to the activation of a compensatory mechanism, which is consistent with a previous study showing an increased number of connector hubs in MCI subjects compared with normal controls (47). It is interesting to note that although the group comparison of morphology changes between EMCI and CN in the ANCOVA did not reach statistical significance, the association between the NBM and their innervated regions allowed us to distinguish EMCI from normal aging subjects.

### The Association Between Brain Structural Changes and Neurocognitive Performance

#### The Effect of APOE-ε4 on the Association Between Brain Atrophy and Neurocognitive Performance

We observed a significant *APOE*-ε4 effect on the correlation between regional brain volumetric changes and cognitive performance in patients with EMCI and AD, with *APOE*-ε4 carriers showing a stronger correlation than non-carriers (Figure 6). The observed interaction effect may be indicative of the effect of the ε4 allele on brain morphology, which may lead to a distinctive cerebral organization supporting cognitive functioning during disease progression (9). The stronger correlation between brain atrophy and cognitive performance for *APOE*-ε4 carriers may imply that *APOE*-ε4 carriers rely on the compensatory brain system to achieve memory performance equivalent to non-carriers (9). This may represent a proxy for available brain reserve to support the cognitive performance.

#### Group Difference in the Association Between Brain Atrophy and Neurocognitive Performance

We found a significant interaction effect between the disease group and regional brain morphology on the neurocognitive performance in SMC, EMCI, and AD subjects. EMCI and AD subjects exhibited a stronger correlation between the left amygdala and MEM than CN subjects did. SMC and AD subjects exhibited a stronger correlation between the left amygdala and LAN than CN subjects did (Figure 7). A previous study has shown that patients with SMC, a population at risk for preclinical AD, exhibit a smaller volume of basal forebrain subdivisions than CN (48). The group comparison of neurocognitive and morphology changes between SMC and CN in our study did not reach a statistical significance. However, the correlation between the cholinergic region and neurocognitive performance remarkably distinguished SMC from CN. Our results provide evidence of the potential involvement of the cholinergic pathway in SMC. AD exhibited a stronger correlation between the cholinergic brain region and neurocognitive performance than CN (memory and language). A compensatory mechanism in AD pathology, as mentioned, possibly reflecting the dysregulation between brain morphology and regional neural functioning is a plausible hypothesis.

### Limitation

This study has some limitations. First, our results are derived from a cross-sectional study, which prevents tracking the time course of the brain changes observed in the disease trajectory in this study. Our results do not represent individual longitudinal changes. Longitudinal data analysis is required for the prediction of the disease progression from one stage to other. However, in the present cross-sectional study, we showed that the combined neurocognitive and volumetric measurements in the cholinergic circuitry yielded a high accuracy in classifying AD from CN. Our results support that the automated classification method using volumetric changes in the cholinergic pathway can possibly facilitate and improve diagnosis. Second, even though the quality of image processing, such as registration, was carefully evaluated, the present study is limited by the indirect nature of volumetric measurements on MRI for cholinergic degeneration. Studies with a larger number of subjects with histopathological examinations should be performed in the future to validate the results.

## Conclusion

Injured brain structures exhibit different morphometric features at various pathological stages. The observed association of *APOE*-ε4 with the brain morphology in the cholinergic pathway and neurocognitive functioning for patients with EMCI, LMCI, and AD can be valuable for disease monitoring.

## Data Availability Statement

The datasets presented in this study can be found in online repositories. The names of the repository/repositories and accession number(s) can be found at: adni.loni.usc.edu.

## Ethics Statement

The studies involving human participants were reviewed and approved by the Alzheimer's Disease Neuroimaging Initiative (ADNI) database (adni.loni.usc.edu). The patients/participants provided their written informed consent to participate in this study.

## Author Contributions

Y-LL and H-SL wrote the main manuscript text and performed the data analysis on the data. Y-LL, C-YC, and H-SL planned and carried out the study. KC performed the machine learning analysis. T-WL, C-WT, H-HL, and L-WK provided intellectual input toward the analysis and interpretation of the MRI data. C-YC and H-SL provided all authors with overall direction in this project. All authors contributed to the article and approved the submitted version.

## Funding

This work was supported by Taipei Medical University Hospital (grants 105TMU-TMUH-05, 106TMU-TMUH-21, and 107TMU-TMUH-09) and Ministry of Science and Technology, R.O.C (grant MOST108-2221-E-038-007-MY2).

## Conflict of Interest

H-HL was employed by the company Rotary Trading Co., Ltd. The remaining authors declare that the research was conducted in the absence of any commercial or financial relationships that could be construed as a potential conflict of interest.

## Publisher's Note

All claims expressed in this article are solely those of the authors and do not necessarily represent those of their affiliated organizations, or those of the publisher, the editors and the reviewers. Any product that may be evaluated in this article, or claim that may be made by its manufacturer, is not guaranteed or endorsed by the publisher.
