# Supplementary material for: The Effect of the APOE-ε4 Allele on the Cholinergic Circuitry for Subjects With Different Levels of Cognitive Impairment
Source: Front Neurol. 2021 Oct 13;12:651388. doi: 10.3389/fneur.2021.651388 (PMC8548434; doi:10.3389/fneur.2021.651388)
Supplement: Supplementary file 1 [file Data_Sheet.docx]

**Supplementary materials and methods**

The detailed study information about the full eligibility criteria for the ADNI is available at [ADNI Study Documents (http://adni.loni.usc.edu/methods/documents/)](http://adni.loni.usc.edu/methods/documents/). The study participants were classified as either cognitively normal (CN), significant memory concern (SMC), early mild cognitive impairment (EMCI), late mild cognitive impairment (LMCI), and Alzheimer's disease (AD). CN subjects had a [Mini-Mental State Examination](https://www.sciencedirect.com/topics/medicine-and-dentistry/mini-mental-state-examination) score (MMSE) ranging from 24 to 30, a [Clinical Dementia Rating](https://www.sciencedirect.com/topics/medicine-and-dentistry/clinical-dementia-rating) (CDR) of 0, and a Memory Box score must be 0, and the education-adjusted cut-off point on the Weschler Memory Scale-Revised Logical Memory II subscale (Delayed Paragraph Recall, Paragraph A only) was ≥9 for 16 or more years of education, ≥5 for 8-15 years of education, and ≥3 for 0-7 years of education, and the absence of significant levels of impairment in other cognitive functions, or activities of daily living, as well as the absence of dementia. Subjects with SMC must have self-reported memory concern and a Cognitive Change Index score (CCI-S) greater than or equal to 16, a [Mini-Mental State Examination](https://www.sciencedirect.com/topics/medicine-and-dentistry/mini-mental-state-examination) score (MMSE) ranging from 24 to 30, a [Clinical Dementia Rating](https://www.sciencedirect.com/topics/medicine-and-dentistry/clinical-dementia-rating) (CDR) of 0, and a Memory Box score of 0, and the education-adjusted cut-off point on the Weschler Memory Scale-Revised Logical Memory II subscale (Delayed Paragraph Recall, Paragraph A only) was ≥9 for 16 or more years of education, ≥5 for 8-15 years of education, and ≥3 for 0-7 years of education, and the absence of significant levels of impairment in other cognitive functions, or activities of daily living, and an absence of dementia. Subjects with EMCI had a [Mini-Mental State Examination](https://www.sciencedirect.com/topics/medicine-and-dentistry/mini-mental-state-examination) score (MMSE) ranging from 24 to 30 inclusive, a [Clinical Dementia Rating](https://www.sciencedirect.com/topics/medicine-and-dentistry/clinical-dementia-rating) (CDR) of 0.5, and a Memory Box score must be at least 0.5, and the education-adjusted cut-off point on the Weschler Memory Scale-Revised Logical Memory II subscale (Delayed Paragraph Recall, Paragraph A only) was 9-11 for 16 or more years of education, 5-9 for 8-15 years of education, and 3-6 for 0-7 years of education, as well as the absence of significant levels of impairment in other cognitive functions, essentially preserved daily living activities and the absence of dementia. Subjects with LCMI had a [Mini-Mental State Examination](https://www.sciencedirect.com/topics/medicine-and-dentistry/mini-mental-state-examination) score (MMSE) ranging from 24 to 30 inclusive, a [Clinical Dementia Rating](https://www.sciencedirect.com/topics/medicine-and-dentistry/clinical-dementia-rating) (CDR) of 0.5, and a Memory Box score must be at least 0.5, and the education-adjusted cut-off point on the Weschler Memory Scale-Revised Logical Memory II subscale (Delayed Paragraph Recall, Paragraph A only) was ≤8 for 16 or more years of education, ≤4 for 8-15 years of education, and ≤2 for 0-7 years of education, as well as the absence of significant levels of impairment in other cognitive functions, essentially preserved daily living activities and the absence of dementia. Subjects with AD subjects had a [Mini-Mental State Examination](https://www.sciencedirect.com/topics/medicine-and-dentistry/mini-mental-state-examination) score (MMSE) ranging from 20 to 26 inclusive, a [Clinical Dementia Rating](https://www.sciencedirect.com/topics/medicine-and-dentistry/clinical-dementia-rating) (CDR) of 0.5 or 1.0, and the education-adjusted cut-off point on the Weschler Memory Scale-Revised Logical Memory II subscale (Delayed Paragraph Recall, Paragraph A only) was ≤8 for 16 or more years of education, ≤4 for 8-15 years of education, and ≤2 for 0-7 years of education, a diagnosis of probable AD based on the National Institute of Neurological and Communicative Diseases and Stroke and the Alzheimer's Disease and Related Disorders Association (NINCDS-ADRDA) criteria^1^.

**Reference**

1. McKhann G, Drachman D, Folstein M, Katzman R, Price D, Stadlan EM. Clinical diagnosis of alzheimer's disease: Report of the nincds‐adrda work group* under the auspices of department of health and human services task force on alzheimer's disease. *Neurology*. 1984;34:939-939

**Supplementary tables**

**Table S1** Summary of non-accelerated MRI protocols for 3D T1-weighted images in ADNI-2.

| Sequence  (Manufacturer) | TR  (ms) | TE  (ms) | Flip angle  (°) | Slice thickness  (mm) | Matrix size  (XYZ; pixels) |
| --- | --- | --- | --- | --- | --- |
| IR-SPGR  (GE) | 6.984 | 2.848 | 11 | 1.2 | 256*256*196 |
| IR-FSPGR (GE) | 9.211 | 3.907 | 8 | 1.2 | 256*256*166 |
| MPRAGE (Philips) | 6.805 | 3.160 | 9 | 1.2 | 256*256*170 |
| MPRAGE (Siemens) | 2300 | 2.980 | 9 | 1.2 | 240*256*176 |

IR-SPGR: inversion recovery spoiled gradient echo, IR-FSPGR: inversion recovery fast spoiled gradient echo, MPRAGE: magnetization prepared rapid acquisition gradient echo, TR: repetition time, TE: echo time

**Table S2** Group effect of MRI volume measurement using 5 × 2 ANCOVA (five diagnostic groups × APOE-ε4 carrier status) (FWE‐corrected, p < 0.05).

| Region |  | P_FWE_ | k_E_ | MNI Coordinates | | |
| --- | --- | --- | --- | --- | --- | --- |
|  |  |  |  | ***x*** | ***y*** | ***z*** |
| Amygdala | Left | 1.52×10^–6^ | 809 | -25.5 | -10.5 | -13.5 |
|  | Right | 4.70×10^–6^ | 711 | 21 | -4.5 | -13.5 |
| Ch123 | Left | 0.002 | 62 | -7.5 | -3 | -10.5 |
|  | Right | 0.003 | 56 | 7.5 | -1.5 | -10.5 |
| NBM | Left | 8.01×10^–6^ | 101 | -18 | -6 | -10.5 |
|  | Right | 8.94×10^–5^ | 68 | 18 | -6 | -10.5 |

**Table S3** Main effect of APOE-ε4 of MRI volume measurement using 5 × 2 ANCOVA (five diagnostic groups × APOE-ε4 carrier status) (FWE‐corrected, p < 0.05).

| Region |  | P_FWE_ | k_E_ | MNI Coordinates | | |
| --- | --- | --- | --- | --- | --- | --- |
|  |  |  |  | ***x*** | ***y*** | ***z*** |
| Amygdala | Left | 0.0004 | 367 | -22.5 | -9 | -15 |
| Amygdala | Right | 0.0005 | 340 | 24 | -9 | -13.5 |
| NBM | Left | 0.008 | 13 | -16.5 | -7.5 | -10.5 |
| NBM | Right | 0.023 | 5 | 18 | -6 | -10.5 |

**Table S4** Significant interaction effect of APOE-ε4×group in the voxel-based morphometry 5 × 2 ANCOVA (five diagnostic groups × APOE-ε4 carrier status) analysis. (FWE‐corrected, p < 0.05).

| Group comparison | Region |  | P_FWE_ | k_E_ | MNI Coordinates | | |
| --- | --- | --- | --- | --- | --- | --- | --- |
|  |  |  |  |  | ***x*** | ***y*** | ***z*** |
| LMCI vs. CN | Amygdala | Left | 4.03×10^–6^ | 796 | -25.5 | -10.5 | -15 |
|  |  | Right | 3.26×10^–5^ | 643 | 24 | -9 | -15 |
|  | NBM | Left | 2.21×10^–5^ | 65 | -21 | -7.5 | -7.5 |
|  |  | Right | 1.98×10^–4^ | 57 | 24 | -9 | -6 |
| AD vs. CN | Amygdala | Left | 0.007 | 497 | -28.5 | -7.5 | -22.5 |
|  |  | Right | 0.004 | 508 | 18 | -4.5 | -16.5 |

**Table S5a** Group comparison of APOE-ε4 effect on global cognitive composite scores and volumetric changes in cognitively normal (CN) subjects.

|  |  | Non-carriers  (n=119) | APOE ε3-ε4 (n=42) | APOE ε4-ε4 (n=6) | P-value |
| --- | --- | --- | --- | --- | --- |
| MEM | mean±  SD | 1.102±  0.603 | 1.038±  0.537 | 0.831±  0.604 | 0.265 |
| EF | mean±  SD | 0.928±  0.801 | 0.931±  0.760 | 0.424±  0.598 | 0.334 |
| LAN | mean±  SD | 0.876±  0.741 | 0.920±  0.608 | 1.093±  0.542 | 0.701 |
| VS | mean±  SD | 0.289±  0.592 | 0.163±  0.520 | 0.411±  0.460 | 0.585 |
| CH123_L | mean±  SD | 0.337±  0.052 | 0.336±  0.049 | 0.314±  0.025 | 0.595 |
| CH123_R | mean±  SD | 0.364±  0.053 | 0.362±  0.047 | 0.342±  0.036 | 0.608 |
| NBM_L | mean±  SD | 0.130±  0.018 | 0.130±  0.019 | 0.125±  0.014 | 0.875 |
| NBM_R | mean±  SD | 0.111±  0.016 | 0.112±  0.016 | 0.102±  0.014 | 0.390 |
| Amy_L | mean±  SD | 0.588±  0.072 | 0.583±  0.072 | 0.581±  0.087 | 0.984 |
| Amy_R  Group differences in global cognitive composite scores were assessed with a one-way analysis of covariance (ANCOVA) test adjusted for age, sex, edu, and TIV. MEM: memory function, EF: executive function, LAN: language, and VS: visuospatial functioning. Non-carriers: (ε2-ε3, ε2-ε2, ε3-ε3), APOE-ε4 heterozygotes: (ε3-ε4), APOE-ε4 homozygotes: (ε4-ε4), NBM: nucleus basalis of Meynert, Amy: amygdala. *P<0.05. | mean±  SD | 0.608±  0.073 | 0.600±  0.061 | 0.600±  0.066 | 0.951 |

**Table S5b** Group comparison of APOE-ε4 effect on global cognitive composite scores and volumetric changes in subjects with significant memory concern (SMC).

|  |  | Non-carriers  (n=64) | APOE ε3-ε4 (n=31) | APOE ε4-ε4 (n=1) | P-value |
| --- | --- | --- | --- | --- | --- |
| MEM | mean±  SD | 1.092±  0.554 | 1.099±  0.619 | 0.616±  . | 0.538 |
| EF | mean±  SD | 0.778±  0.831 | 0.857±  0.889 | 0.326±  . | 0.890 |
| LAN | mean±  SD | 0.716±  0.661 | 0.816±  0.747 | 0.344±  . | 0.905 |
| VS | mean±  SD | 0.182±  0.599 | 0.238±  0.633 | 0.706±  . | 0.520 |
| CH123_L | mean±  SD | 0.337±  0.047 | 0.335±  0.046 | 0.363±  . | 0.712 |
| CH123_R | mean±  SD | 0.365±  0.051 | 0.369±  0.043 | 0.362±  . | 0.952 |
| NBM_L | mean±  SD | 0.131±  0.02 | 0.131±  0.013 | 0.139±  . | 0.973 |
| NBM_R | mean±  SD | 0.111±  0.017 | 0.113±  0.014 | 0.112±  . | 0.783 |
| Amy_L | mean±  SD | 0.589±  0.078 | 0.588±  0.052 | 0.632±  . | 0.880 |
| Amy_R | mean±  SD | 0.602±  0.08 | 0.603±  0.05 | 0.632±  . | 0.988 |

Group differences in global cognitive composite scores were assessed with a one-way analysis of covariance (ANCOVA) test adjusted for age, sex, edu, and TIV. MEM: memory function, EF: executive function, LAN: language, and VS: visuospatial functioning. Non-carriers: (ε2-ε3, ε2-ε2, ε3-ε3), APOE-ε4 heterozygotes: (ε3-ε4), APOE-ε4 homozygotes: (ε4-ε4), NBM: nucleus basalis of Meynert, Amy: amygdala. *P<0.05.

**Table S5c** Group comparison of APOE-ε4 effect on global cognitive composite scores and volumetric changes in subjects with early mild cognitive impairment (EMCI).

|  |  | Non-carriers  (n=79) | APOE ε3-ε4 (n=55) | APOE ε4-ε4 (n=12) | P-value |
| --- | --- | --- | --- | --- | --- |
| MEM | mean±  SD | 0.670±  0.630 | 0.527±  0.609 | 0.348±  0.399 | 0.067 |
| EF | mean±  SD | 0.693±  0.811 | 0.438±  0.861 | 0.285±  0.838 | 0.054 |
| LAN | mean±  SD | 0.593±  0.753 | 0.413±  0.755 | 0.298±  0.756 | 0.152 |
| VS | mean±  SD | 0.113±  0.725 | -0.056±  0.710 | 0.087±  0.632 | 0.408 |
| CH123_L | mean±  SD | 0.323±  0.047 | 0.331±  0.044 | 0.348±  0.073 | 0.652 |
| CH123_R | mean±  SD | 0.349±  0.050 | 0.357±  0.047 | 0.380±  0.071 | 0.494 |
| NBM_L | mean±  SD | 0.127±  0.020 | 0.128±  0.017 | 0.122±  0.018 | 0.854 |
| NBM_R | mean±  SD | 0.110±  0.016 | 0.109±  0.014 | 0.107±  0.016 | 0.578 |
| Amy_L | mean±  SD | 0.560±  0.088 | 0.572±  0.081 | 0.562±  0.093 | 0.846 |
| Amy_R | mean±  SD | 0.583±  0.085 | 0.591±  0.081 | 0.590±  0.104 | 0.977 |

Group differences in global cognitive composite scores were assessed with a one-way analysis of covariance (ANCOVA) test adjusted for age, sex, edu, and TIV. MEM: memory function, EF: executive function, LAN: language, and VS: visuospatial functioning. Non-carriers: (ε2-ε3, ε2-ε2, ε3-ε3), APOE-ε4 heterozygotes: (ε3-ε4), APOE-ε4 homozygotes: (ε4-ε4), NBM: nucleus basalis of Meynert, Amy: amygdala. *P<0.05.

**Table S5d** Group comparison of APOE-ε4 effect on global cognitive composite scores and volumetric changes in subjects with late mild cognitive impairment (LMCI).

|  |  | Non-carriers  (n=57) | APOE ε3-ε4 (n=55) | APOE ε4-ε4 (n=26) | P-value |
| --- | --- | --- | --- | --- | --- |
| MEM | mean±  SD | 0.180±  0.636 | -0.05±  0.613 | -0.310±  0.558 | **0.001*** |
| EF | mean±  SD | 0.263±  0.936 | 0.082±  0.889 | 0.237±  0.696 | 0.512 |
| LAN | mean±  SD | 0.333±  0.766 | 0.036±  0.836 | 0.175±  0.745 | 0.276 |
| VS | mean±  SD | -0.177±  0.757 | -0.131±  0.775 | -0.164±  0.671 | 0.902 |
| CH123_L | mean±  SD | 0.319±  0.046 | 0.321±  0.058 | 0.314±  0.049 | 0.499 |
| CH123_R | mean±  SD | 0.346±  0.046 | 0.349±  0.058 | 0.346±  0.055 | 0.641 |
| NBM_L | mean±  SD | 0.124±  0.017 | 0.114±  0.020 | 0.111±  0.018 | **0.001*** |
| NBM_R | mean±  SD | 0.108±  0.016 | 0.098±  0.017 | 0.098±  0.015 | **0.002*** |
| Amy_L | mean±  SD | 0.557±  0.079 | 0.499±  0.101 | 0.470±  0.979 | **0.001*** |
| Amy_R | mean±  SD | 0.573±  0.089 | 0.521±  0.099 | 0.502±  0.082 | **0.001*** |

Group differences in global cognitive composite scores were assessed with a one-way analysis of covariance (ANCOVA) test adjusted for age, sex, edu, and TIV. MEM: memory function, EF: executive function, LAN: language, and VS: visuospatial functioning. Non-carriers: (ε2-ε3, ε2-ε2, ε3-ε3), APOE-ε4 heterozygotes: (ε3-ε4), APOE-ε4 homozygotes: (ε4-ε4), NBM: nucleus basalis of Meynert, Amy: amygdala. *P<0.05.

**Table S5e** Post hoc multiple comparisons of APOE-ε4 effect on global cognitive composite scores and volumetric changes in LMCI.

| Comparison Group | Mean Difference | Std. Error | Sig. |
| --- | --- | --- | --- |
| MEM | | | |
| Non-carriers  **-** APOE ε3-ε4 | 0.257 | 0.109 | 0.061 |
| Non-carriers  **-** APOE ε4-ε4 | **0.542*** | 0.138 | **0.001*** |
| APOE ε3-ε4 –  APOE ε4-ε4 | 0.285 | 0.138 | 0.125 |
| NBM_L | | | |
| Non-carriers  **-** APOE ε3-ε4 | **0.001*** | 0.003 | **0.016*** |
| Non-carriers  **-** APOE ε4-ε4 | **0.014*** | 0.004 | **0.002*** |
| APOE ε3-ε4 –  APOE ε4-ε4 | 0.005 | 0.004 | 0.652 |
| NBM_R | | | |
| Non-carriers  **-** APOE ε3-ε4 | **0.010*** | 0.003 | **0.004*** |
| Non-carriers  **-** APOE ε4-ε4 | **0.010*** | 0.004 | **0.021*** |
| APOE ε3-ε4 –  APOE ε4-ε4 | 0.000 | 0.004 | 1.000 |
| Amy_L | | | |
| Non-carriers  **-** APOE ε3-ε4 | **0.054*** | 0.015 | **0.002*** |
| Non-carriers  **-** APOE ε4-ε4 | **0.098*** | 0.019 | **0.001*** |
| APOE ε3-ε4 –  APOE ε4-ε4 | 0.044 | 0.020 | 0.076 |
| Amy_R | | | |
| Non-carriers  **-** APOE ε3-ε4 | **0.048*** | 0.015 | **0.006*** |
| Non-carriers  **-** APOE ε4-ε4 | **0.083*** | 0.019 | **0.001*** |
| APOE ε3-ε4 –  APOE ε4-ε4 | 0.034 | 0.020 | 0.241 |
| *. The mean difference is significant at the 0.05 level in Tukey’s HSD test. | | | |

**Table S5f** Group comparison of APOE-ε4 effects on global cognitive composite scores and volumetric changes in subjects with Alzheimer’s disease (AD).

|  |  | Non-carriers  (n=34) | APOE ε3-ε4 (n=60) | APOE ε4-ε4 (n=27) | P-value |
| --- | --- | --- | --- | --- | --- |
| MEM | mean±  SD | -0.872±  0.483 | -0.903±  0.632 | -0.846±  0.527 | 0.850 |
| EF | mean±  SD | -0.882±  0.905 | -0.846±  0.944 | -0.603±  0.844 | 0.305 |
| LAN | mean±  SD | -0.904±  0.814 | -0.794±  1.086 | -0.474±  0.825 | 0.204 |
| VS | mean±  SD | -0.574±  0.720 | -0.464±  0.977 | -0.660±  1.097 | 0.710 |
| CH123_L | mean±  SD | 0.314±  0.049 | 0.301±  0.043 | 0.312±  0.041 | 0.704 |
| CH123_R | mean±  SD | 0.339±  0.049 | 0.352±  0.047 | 0.336±  0.049 | 0.697 |
| NBM_L | mean±  SD | 0.111±  0.019 | 0.103±  0.019 | 0.105±  0.016 | 0.218 |
| NBM_R | mean±  SD | 0.097±  0.016 | 0.090±  0.015 | 0.092±  0.019 | 0.158 |
| Amy_L | mean±  SD | 0.485±  0.095 | 0.433±  0.086 | 0.447±  0.080 | **0.021*** |
| Amy_R | mean±  SD | 0.513±  0.960 | 0.457±  0.085 | 0.461±  0.072 | **0.007*** |

Group differences in global cognitive composite scores were assessed with a one-way analysis of covariance (ANCOVA) test adjusted for age, sex, edu, and TIV. MEM: memory function, EF: executive function, LAN: language, and VS: visuospatial functioning. Non-carriers: (ε2-ε3, ε2-ε2, ε3-ε3), APOE-ε4 heterozygotes: (ε3-ε4), APOE-ε4 homozygotes: (ε4-ε4), NBM: nucleus basalis of Meynert, Amy: amygdala. *P<0.05.

**Table S5g** Post hoc multiple comparisons of APOE-ε4 effects on global cognitive composite scores and volumetric changes in AD.

| Comparison Group | Mean Difference | Std. Error | Sig. |
| --- | --- | --- | --- |
| Amy_L | | | |
| Non-carriers  **-** APOE ε3-ε4 | **0.042*** | 0.016 | **0.030*** |
| Non-carriers  **-** APOE ε4-ε4 | 0.044 | 0.019 | 0.074 |
| APOE ε3-ε4 –  APOE ε4-ε4 | 0.002 | 0.017 | 1.000 |
| Amy_R | | | |
| Non-carriers  **-** APOE ε3-ε4 | **0.046*** | 0.016 | **0.015*** |
| Non-carriers  **-** APOE ε4-ε4 | 0.054 | 0.019 | 0.019 |
| APOE ε3-ε4 –  APOE ε4-ε4 | 0.008 | 0.017 | 1.000 |
| *. The mean difference is significant at the 0.05 level in Tukey’s HSD test. | | | |

**Table S6** Results of classification accuracy for all features (All) and excluding APOE-ε4 status (All- APOE-ε4), neurocognitive performance (All-neurocognitive performance) or VBM (All-neurocognitive performance) measurements at a time from the overall model.

| **Classification** | All | All- APOE-ε4 | All-neurocognitive performance | All-VBM measurements |
| --- | --- | --- | --- | --- |
| SMC-CN  EMCI-CN  LMCI-CN  AD-CN | 0.468  0.648  0.804  0.966 | 0.418  0.660  0.793  0.977 | 0.570  0.628  0.793  0.897 | 0.582  0.628  0.783  0.783 |

All: all features, All- APOE-ε4: all features exclude APOE-ε4 status, All-VBM measurements: all features exclude VBM measurements.

**Table S7** Linear regression analysis with effects of APOE-ε4 and interaction of APOE-ε4 × volumetric measurements in modulating the neurocognitive performance using the regression model: neurocognitive scores = NBM_L + NBM_R + amygdala_L + amygdala_R + APOE-ε4 + (APOE-ε4 × NBM_L) + (APOE-ε4 × NBM_R) + (APOE-ε4 × amygdala_L) + (APOE-ε4 × amygdala_R) + age + sex + edu + TIV

|  |  | CN | SMC | EMCI | LMCI | AD |
| --- | --- | --- | --- | --- | --- | --- |
| MEM | F  (P-value) | 12.24  (<0.0005) | 9.28  (<0.0005) | 22.56  (<0.0005) | 11.73  (<0.0005) | 16.44  (<0.0005) |
|  | NBM_L  β (P-value) | NS | NS | NS | NS | NS |
|  | NBM_R  β (P-value) | NS | NS | NS | NS | NS |
|  | Amy_L  β (P-value) | NS | NS | 2.36  (<0.0005) | 1.80  (0.001) | 3.98  (<0.0005) |
|  | Amy_R  β (P-value) | NS | NS | NS | NS | 1.78  (0.021) |
|  | APOE-ε4 status  β (P-value) | NS | NS | NS | -0.21  (0.049) | NS |
|  | APOE-ε4 × NBM_L  β (P-value) | NS | NS | NS | NS | NS |
|  | APOE-ε4 × NBM_R  β (P-value) | NS | NS | NS | NS | NS |
|  | APOE-ε4 × Amy_L  β (P-value) | NS | NS | **0.35**  **(*0.015)** | NS | NS |
|  | APOE-ε4 × Amy_R  β (P-value) | NS | NS | NS | NS | NS |
|  | Age  β (P-value) | -0.03  (<0.0005) | -0.03  (0.004) | -0.03  (<0.0005) | NS | NS |
|  | Sex  β (P-value) | -0.35  (<0.0005) | -0.42  (<0.0005) | -0.42  (<0.0005) | -0.42  (<0.0005) | NS |
|  | Edu  β (P-value) | 0.04  (0.024) | 0.06  (0.007) | NS | NS | NS |
|  | TIV  β (P-value) | NS | NS | NS | NS | NS |
| EF | F  (P-value) | 22.98  (<0.0005) | 7.38  (0.001) | 20.41  (<0.0005) | 8.95  (<0.0005) | 4.03 (0.047) |
|  | NBM_L  β (P-value) | 11.00  (0.001) | NS | NS | NS | NS |
|  | NBM_R  β (P-value) | NS | NS | NS | NS | NS |
|  | Amy_L  β (P-value) | NS | 2.68  (0.03) | NS | NS | 1.84  (0.047) |
|  | Amy_R  β (P-value) | NS | NS | NS | 1.66  (0.03) | NS |
|  | APOE-ε4 status  β (P-value) | NS | NS | NS | NS | NS |
|  | APOE-ε4 × NBM_L  β (P-value) | NS | NS | NS | NS | NS |
|  | APOE-ε4 × NBM_R  β (P-value) | NS | NS | NS | NS | NS |
|  | APOE-ε4 × Amy_L  β (P-value) | NS | NS | NS | NS | NS |
|  | APOE-ε4 × Amy_R  β (P-value) | NS | NS | NS | NS | NS |
|  | Age  β (P-value) | -0.04 (<0.0005) | -0.034  (0.03) | -0.05  (<0.0005) | -0.03  (0.006) | NS |
|  | Sex  β (P-value) | NS | NS | NS | NS | NS |
|  | Edu  β (P-value) | NS | NS | NS | 0.07 (0.014) | NS |
|  | TIV  β (P-value) | NS | NS | NS | NS | NS |
| LAN | F  (P-value) | 8.971 (<0.0005) | 8.811 (<0.0005) | 15.67 (<0.0005) | 15.33 (<0.0005) | 13.66 (<0.0005) |
|  | NBM_L  β (P-value) | NS | NS | 11.12  (0.001) | NS | NS |
|  | NBM_R  β (P-value) | NS | NS | NS | NS | NS |
|  | Amy_L  β (P-value) | NS | 4.03 (<0.0005) | NS | 2.31 (<0.0005) | 7.30 (<0.0005) |
|  | Amy_R  β (P-value) | NS | NS | NS | NS | NS |
|  | APOE-ε4 status  β (P-value) | NS | NS | NS | NS | NS |
|  | APOE-ε4 × NBM_L  β (P-value) | NS | NS | NS | NS | **3.38**  **(0.03*)** |
|  | APOE-ε4 × NBM_R  β (P-value) | NS | NS | NS | NS | NS |
|  | APOE-ε4 × Amy_L  β (P-value) | NS | NS | NS | NS | NS |
|  | APOE-ε4 × Amy_R  β (P-value) | NS | NS | NS | NS | NS |
|  | Age  β (P-value) | -0.03  (0.003) | NS | -0.03 (<0.0005) | NS | NS |
|  | Sex  β (P-value) | NS | -0.30  (0.032) | NS | NS | NS |
|  | Edu  β (P-value) | 0.06  (0.005) | 0.07  (0.005) | NS | 0.09 (<0.0005) | NS |
|  | TIV  β (P-value) | NS | NS | NS | NS | NS |
| VS | F  (P-value) | 7.377  (0.001) | NS | 5.68  (0.02) | NS | 6.17  (0.003) |
|  | NBM_L  β (P-value) | 5.09  (0.032) | NS | 7.64  (0.02) | NS | NS |
|  | NBM_R  β (P-value) | NS | NS | NS | NS | NS |
|  | Amy_L  β (P-value) | NS | NS | NS | NS | NS |
|  | Amy_R  β (P-value) | NS | NS | NS | NS | NS |
|  | APOE-ε4 status  β (P-value) | NS | NS | NS | NS | NS |
|  | APOE-ε4 × NBM_L  β (P-value) | NS | NS | NS | NS | NS |
|  | APOE-ε4 × NBM_R  β (P-value) | NS | NS | NS | NS | NS |
|  | APOE-ε4 × Amy_L  β (P-value) | NS | NS | NS | NS | NS |
|  | APOE-ε4 × Amy_R  β (P-value) | NS | NS | NS | NS | NS |
|  | Age  β (P-value) | NS | NS | NS | NS | 0.023  (0.024) |
|  | Sex  β (P-value) | NS | NS | NS | NS | NS |
|  | Edu  β (P-value) | 0.05  (0.003) | NS | NS | NS | 0.09  (0.006) |
|  | TIV  β (P-value) | NS | NS | NS | NS | NS |

NS: not significant, *Significant interaction term of APOE-ε4 × volumetric measurements indicated that the relationship between the volumetric changes in the cholinergic regions and neurocognitive performance is different in APOE-ε4 carriers and noncarriers.

**Table S8** Linear regression analysis with effects of group and interaction of group × volumetric measurements in modulating the neurocognitive performance using the regression model:

neurocognitive scores = NBM_L + NBM_R + amygdala_L + amygdala_R + group + (group × NBM_L) + (group × NBM_R) + (group × amygdala_L) + (group × amygdala_R) + age + sex + edu + TIV

|  |  | SMC vs. CN | eMCI vs. CN | lMCI vs. CN | AD vs. CN |
| --- | --- | --- | --- | --- | --- |
| **MEM** | Model F  (P-value) | 23.61  (<0.0005) | 40.12  (<0.0005) | 72.94  (<0.0005) | 325.51  (<0.0005) |
|  | Group  β (P-value) | NS | -1.06  (<0.0005) | -0.33  (<0.0005) | -2.40  (<0.0005) |
|  | NBM_L  β (P-value) | NS | NS | NS | NS |
|  | NBM_R  β (P-value) | NS | NS | 8.28  (<0.0005) | NS |
|  | Amy_L  β (P-value) | NS | NS | NS | NS |
|  | Amy_R  β (P-value) | NS | NS | NS | NS |
|  | Group×NBM_L  β (P-value) | NS | NS | NS | NS |
|  | Group×NBM_R  β (P-value) | NS | NS | NS | NS |
|  | Group×Amy_L  β (P-value) | NS | **1.006**  **(*<0.0005)** | NS | **1.49**  **(*<0.0005)** |
|  | Group×Amy_R  β (P-value) | NS | NS | NS | NS |
|  | Age  β (P-value) | -0.03  (<0.0005) | -0.03  (<0.0005) | -0.02  (0.001) | -0.32  (<0.0005) |
|  | Sex  β (P-value) | -0.34  (<0.0005) | -0.39  (<0.0005) | -0.42  (<0.0005) | NS |
|  | Edu  β (P-value) | 0.045  (0.001) | 0.035  (0.003) | 0.04  (0.002) | NS |
|  | TIV  β (P-value) | NS | NS | NS | NS |
| **EF** | Model F  (P-value) | 16.48  (<0.0005) | 26.75  (<0.0005) | 30.03  (<0.0005) | 87.97  (<0.0005) |
|  | Group  β (P-value) | 12.00 (<0.0005) | -0.42 (<0.0005) | -0.22 (<0.0005) | -1.45 (<0.0005) |
|  | NBM_L  β (P-value) | NS | 8.54 (<0.0005) | NS | 9.44 (<0.0005) |
|  | NBM_R  β (P-value) | NS | NS | NS | NS |
|  | Amy_L  β (P-value) | NS | NS | NS | NS |
|  | Amy_R  β (P-value) | NS | NS | 1.84 (0.001) | NS |
|  | Group×NBM_L  β (P-value) | NS | NS | NS | NS |
|  | Group×NBM_R  β (P-value) | NS | NS | NS | NS |
|  | Group×Amy_L  β (P-value) | NS | NS | NS | NS |
|  | Group×Amy_R  β (P-value) | NS | NS | NS | NS |
|  | Age  β (P-value) | -0.04  (<0.0005) | -0.04  (<0.0005) | -0.04  (<0.0005) | -0.02  (0.022) |
|  | Sex  β (P-value) | NS | NS | NS | NS |
|  | Edu  β (P-value) | NS | 0.042  (0.009) | 0.05  (0.002) | 0.04  (0.022) |
|  | TIV  β (P-value) | NS | NS | NS | NS |
| **LAN** | Model F  (P-value) | 9.762  (<0.0005) | 19.10  (<0.0005) | 32.47  (<0.0005) | 78.15  (<0.0005) |
|  | Group  β (P-value) | -0.82  (0.002) | -0.40  (<0.0005) | -0.21  (<0.0005) | -1.75  (<0.0005) |
|  | NBM_L  β (P-value) | NS | 9.222  (<0.0005) | 7.02  (0.002) | NS |
|  | NBM_R  β (P-value) | NS | NS | NS | NS |
|  | Amy_L  β (P-value) | NS | NS | NS | NS |
|  | Amy_R  β (P-value) | NS | NS | NS | NS |
|  | Group×NBM_L  β (P-value) | NS | NS | NS | NS |
|  | Group×NBM_R  β (P-value) | NS | NS | NS | NS |
|  | Group×Amy_L  β (P-value) | **1.08**  **(*0.01)** | NS | NS | **3.20**  **(*<0.0005)** |
|  | Group×Amy_R  β (P-value) | NS | NS | NS | NS |
|  | Age  β (P-value) | -0.02 (0.01) | -0.02  (<0.0005) | -0.02 (0.003) | -0.02  (0.012) |
|  | Sex  β (P-value) | NS | -0.172 (0.04) | NS | NS |
|  | Edu  β (P-value) | 0.062  (<0.0005) | 0.06  (<0.0005) | 0.07  (<0.0005) | 0.04  (0.02) |
|  | TIV  β (P-value) | NS | NS | NS | NS |
| **VS** | Model F  (P-value) | 5.535 (0.019) | 8.721  (<0.0005) | 20.67  (<0.0005) | 35.62  (<0.0005) |
|  | Group  β (P-value) | NS | NS | -0.14  (<0.0005) | -0.62  (<0.0005) |
|  | NBM_L  β (P-value) | 4.648  (0.019) | 6.80  (0.001) | NS | 5.73  (0.015) |
|  | NBM_R  β (P-value) | NS | NS | NS | NS |
|  | Amy_L  β (P-value) | NS | NS | NS | NS |
|  | Amy_R  β (P-value) | NS | NS | NS | NS |
|  | Group×NBM_L  β (P-value) | NS | NS | NS | NS |
|  | Group×NBM_R  β (P-value) | NS | NS | NS | NS |
|  | Group×Amy_L  β (P-value) | NS | NS | NS | NS |
|  | Group×Amy_R  β (P-value) | NS | NS | NS | NS |
|  | Age  β (P-value) | NS | -0.01  (0.03) | NS | NS |
|  | Sex  β (P-value) | NS | NS | NS | NS |
|  | Edu  β (P-value) | NS | 0.04  (0.006) | 0.05  (0.002) | 0.06  (<0.0005) |
|  | TIV  β (P-value) | NS | NS | NS | NS |

NS: not significant. *Significant interaction term of group × volumetric measurements indicated that the relationship between the volumetric changes in the cholinergic regions and neurocognitive performance is different in disease group and cognitively normal subjects.
